# Supplementary material for: Inhibition of miR-152 during In Vitro Maturation Enhances the Developmental Potential of Porcine Embryos
Source: Animals (Basel). 2020 Dec 4;10(12):2289. doi: 10.3390/ani10122289 (PMC7761803; doi:10.3390/ani10122289)
Supplement: Supplementary file 1 [file animals-10-02289-s001.pdf]

animals MDPI

Table S1. List of primers used for real-time RT-qPCR.

Gene Transcript	Primers 5'–3'	Amplicon Length (bp)	T_{an} (°C)	GenBank Accession Number
HSP90B1	GGA TGG TCA GGC AAC ATG GA<br>ATA CCC TGA CCG CAG TGT TG	225	55	NM_214103
IGF1	TGC TCT CCT TCA CCA GCT CT<br>GCC TCC TCA GAT CAC AGC TC	198	60	NM_214256
IGF1R	ATA CCA GGG CTT GTC CAA CG<br>CGG CTT GTT CTC CTC ACT GT	226	53	NM_214172
IGFBP6	GCG TCC AAG ACA CTG AGA TG<br>CAC TGC CGC TTC CGA TAG AA	142	54	NM_001100190
IGFBP7	TCC ATC GTG ACA CCC CCT AA<br>GCG TCT GAA TGG CCA GGT TA	181	55	NM_001163801
IRS1	TGC CTG ACC AGC AAG ACC ATC<br>ATC CAC CTG CAT CCA AAA CTC	171	60	NM_001244489
PPP1CB	ACT TTC TGG TAA CAC CCT CAC CTG CA<br>CAA ACG TCC CAC TGA CCA GCA TTC	237	55	NM_214184
PTEN	CCA GTC AGA GGC GCT ATG TG<br>TGA ACT TGT CTT CCC GTC GTG	210	60	NM_001143696
H1FOO	GCA AAG GTC AAA GCA GAG GCT<br>TAC CTT CCT GGC TGG TGG TT	231	58	NM_001205063
TUBA1B	AGT TTT CTG AGG CCC GTG AG<br>TGC AGG GCT TAA AGG AAT GGT	130	58	NM_001044544
BAX	AAG CGC ATT GGA GAT GAA CT<br>CGA TCT CGA AGG AAG TCC AG	251	55	XM_003127290
BCL2	GAC AAC ATC GCC CTG TGG AT<br>CAC TTA TGG CCC AGA TAG GCA	209	55	XM_021099593
GJA1	AGT GAT CCT TAC CAC GCC AC<br>CGA TTC TGC TCG GCA CTG TA	227	58	NM_001244212
PAPOLA	ATG ACA GCA GCC TCG ACT TG<br>TGC TTT CGC TCG ATG TAC CC	228	55	XM_021099679
SOD1	AAC ATG GTG GGC CAA AGG AT<br>GTG CGG CCA ATG ATG GAA TG	139	55	NM_001190422
SOD2	GGT GGA GGC CAC ATC AAT CA<br>AAC AAG CGG CAA TCT GCA AG	220	55	NM_214127
H2AFZ	GGC CGT ATT CAT CGA CAC CTG<br>ACC AGC GAT TGT AGC CTT GAT	231	62	NM_001123122
RPS18	GGC CAA CGG TCT GGA TAA CA<br>TCT TCT TGG ACA CAC CCA CG	158	55	NM_213940

T_{an}—annealing temperature

Table S2. Predicted miR-152 target genes.

miRNA ID	miRNA Acc	Refseq	Symbol	Description	diana	microinspector	miranda	mirtarget2	mitarget	nbmirtar	pictar	pita	rna22	rnahybrid	targetscan
hsa-miR-152	MIMAT0000438	NM_000052	ATP7A	ATPase, Cu++ transporting, alpha polypeptide (Menkes syndrome)	0	0	1	0	0	0	0	1	0	1	1
hsa-miR-152	MIMAT0000438	NM_000125	ESR1	estrogen receptor 1	0	0	1	1	0	0	1	1	0	1	1
hsa-miR-152	MIMAT0000438	NM_000138	FBN1	fibrillin 1	0	0	0	1	0	0	0	1	0	1	1
hsa-miR-152	MIMAT0000438	NM_000242	MBL2	mannose-binding lectin (protein C) 2, soluble (opsonic defect)	0	0	1	1	0	0	0	1	0	1	0
hsa-miR-152	MIMAT0000438	NM_000248	MITF	microphthalmia-associated transcription factor	0	0	1	1	0	0	1	1	0	1	1
hsa-miR-152	MIMAT0000438	NM_000297	PKD2	polycystic kidney disease 2 (autosomal dominant)	0	0	1	1	0	0	0	1	0	1	0
hsa-miR-152	MIMAT0000438	NM_000311	PRNP	prion protein (p27-30) (Creutzfeldt-Jakob disease, Gerstmann-Strausler-Scheinker syndrome, fatal familial insomnia)	0	0	1	1	0	0	0	1	0	1	0
hsa-miR-152	MIMAT0000438	NM_000314	PTEN	phosphatase and tensin homolog (mutated in multiple advanced cancers 1)	0	0	1	1	0	0	0	1	0	1	1
hsa-miR-152	MIMAT0000438	NM_000332	ATXN1	ataxin 1	0	0	1	0	0	0	1	1	0	1	1
hsa-miR-152	MIMAT0000438	NM_000441	SLC26A4	solute carrier family 26, member 4	0	0	1	1	0	0	0	1	0	1	0
hsa-miR-152	MIMAT0000438	NM_000527	LDLR	low density lipoprotein receptor (familial hypercholesterolemia)	0	0	1	1	0	0	0	1	0	1	1
hsa-miR-152	MIMAT0000438	NM_000615	NCAM1	neural cell adhesion molecule 1	0	0	1	0	0	0	0	1	0	1	1
hsa-miR-152	MIMAT0000438	NM_000618	IGF1	insulin-like growth factor 1 (somatomedin C)	0	0	1	1	0	0	0	1	0	1	0
hsa-miR-152	MIMAT0000438	NM_000627	LTBP1	latent transforming growth factor beta binding protein 1	0	0	1	0	0	0	1	1	0	1	1
hsa-miR-152	MIMAT0000438	NM_000705	ATP4B	ATPase, H+/K+ exchanging, beta polypeptide	0	0	1	1	0	0	0	1	0	1	0
hsa-miR-152	MIMAT0000438	NM_000757	CSF1	colony stimulating factor 1 (macrophage)	0	0	1	0	0	0	1	1	0	1	1
hsa-miR-152	MIMAT0000438	NM_000899	KITLG	KIT ligand	0	0	0	0	0	0	1	1	0	1	1
hsa-miR-152	MIMAT0000438	NM_000909	NPY1R	neuropeptide Y receptor Y1	0	0	1	1	0	0	0	1	0	1	0
hsa-miR-152	MIMAT0000438	NM_001001396	ATP2B4	ATPase, Ca++ transporting, plasma membrane 4	0	0	1	0	0	0	0	1	0	1	1
hsa-miR-152	MIMAT0000438	NM_001001481	UBE2W	ubiquitin-conjugating enzyme E2W (putative)	0	0	1	0	0	0	1	1	0	1	1
hsa-miR-152	MIMAT0000438	NM_001001557	GDF6	growth differentiation factor 6	0	0	1	0	0	0	1	1	0	1	1
hsa-miR-152	MIMAT0000438	NM_001002909	GPATCH8	G patch domain containing 8	0	0	1	1	0	0	1	1	0	1	1
hsa-miR-152	MIMAT0000438	NM_001003652	SMAD2	SMAD family member 2	0	0	1	0	0	0	0	1	0	1	1
hsa-miR-152	MIMAT0000438	NM_001003694	BRPF1	bromodomain and PHD finger containing, 1	0	0	1	0	0	0	1	1	0	1	1

hsa-miR-152	MIMAT0000438	NM_001004053	A26C3	ANKRD26-like family C, member 3	0	0	1	1	0	0	0	1	0	1	0
hsa-miR-152	MIMAT0000438	NM_001004303	C1orf168	chromosome 1 open reading frame 168	0	0	0	0	0	0	1	1	0	1	1
hsa-miR-152	MIMAT0000438	NM_001004354	NRARP	Notch-regulated ankyrin repeat protein	0	0	1	0	0	0	0	1	0	1	1
hsa-miR-152	MIMAT0000438	NM_001004439	ITGA11	integrin, alpha 11	0	0	0	1	0	0	1	1	0	1	1
hsa-miR-152	MIMAT0000438	NM_001005210	LRRC55	leucine rich repeat containing 55	0	0	1	0	0	0	1	1	0	1	1
hsa-miR-152	MIMAT0000438	NM_001005353	AK3L1	adenylate kinase 3-like 1	0	0	1	1	0	0	0	1	0	1	0
hsa-miR-152	MIMAT0000438	NM_001005366	FBXL10	F-box and leucine-rich repeat protein 10	0	0	1	0	0	0	1	1	0	1	1
hsa-miR-152	MIMAT0000438	NM_001005408	KIAA1787	KIAA1787 protein	0	0	1	0	0	0	1	1	0	1	1
hsa-miR-152	MIMAT0000438	NM_001005415	MARCHF2	membrane-associated ring finger (C3HC4) 2	0	0	0	1	0	0	1	1	0	1	1
hsa-miR-152	MIMAT0000438	NM_001005416	MARCHF2	membrane-associated ring finger (C3HC4) 2	0	0	0	1	0	0	1	1	0	1	0
hsa-miR-152	MIMAT0000438	NM_001005417	B4GALT2	UDP-Gal:betaGlcNAc beta 1,4- galactosyltransferase, polypeptide 2	0	0	1	0	0	0	1	1	0	1	1
hsa-miR-152	MIMAT0000438	NM_001007075	KLHL5	kelch-like 5 (Drosophila)	0	0	1	1	0	0	0	1	0	1	0
hsa-miR-152	MIMAT0000438	NM_001008239	C18orf25	chromosome 18 open reading frame 25	0	0	0	1	0	0	0	1	0	1	1
hsa-miR-152	MIMAT0000438	NM_001008537	KIAA2022	KIAA2022	0	0	1	0	0	0	0	1	0	1	1
hsa-miR-152	MIMAT0000438	NM_001008660	PICALM	phosphatidylinositol binding clathrin assembly protein	0	0	1	0	0	0	0	1	0	1	1
hsa-miR-152	MIMAT0000438	NM_001009569	MLLT10	myeloid/lymphoid or mixed-lineage leukemia (trithorax homolog, Drosophila); translocated to, 10	0	0	1	1	0	0	0	1	0	1	1
hsa-miR-152	MIMAT0000438	NM_001009993	FAM168B	family with sequence similarity 168, member B	0	0	1	1	0	0	0	1	0	1	0
hsa-miR-152	MIMAT0000438	NM_001013439	FXR1	fragile X mental retardation, autosomal homolog 1	0	0	1	1	0	0	0	1	0	1	0
hsa-miR-152	MIMAT0000438	NM_001015051	RUNX2	runt-related transcription factor 2	0	0	1	0	0	0	0	1	0	1	1
hsa-miR-152	MIMAT0000438	NM_001015880	PAPSS2	3''-phosphoadenosine 5''-phosphosulfate synthase 2	0	0	1	1	0	0	0	1	0	1	0
hsa-miR-152	MIMAT0000438	NM_001017523	BTBD11	BTB (POZ) domain containing 11	0	0	1	0	0	0	0	1	0	1	1
hsa-miR-152	MIMAT0000438	NM_001018068	SERBP1	SERPINE1 mRNA binding protein 1	0	0	1	1	0	0	0	1	0	1	0
hsa-miR-152	MIMAT0000438	NM_001018069	SERBP1	SERPINE1 mRNA binding protein 1	0	0	1	1	0	0	0	1	0	1	0
hsa-miR-152	MIMAT0000438	NM_001024649	CANX	calnexin	0	0	1	0	0	0	0	1	0	1	1
hsa-miR-152	MIMAT0000438	NM_001024808	BCL7A	B-cell CLL/lymphoma 7A	0	0	1	0	0	0	0	1	0	1	1
hsa-miR-152	MIMAT0000438	NM_001024843	TNRC6B	trinucleotide repeat containing 6B	0	0	1	1	0	0	0	1	0	1	1
hsa-miR-152	MIMAT0000438	NM_001025300	RAB12	RAB12, member RAS oncogene family	0	0	0	1	0	0	0	1	0	1	1

hsa-miR-152	MIMAT0000438	NM_001025593	ARFIP1	ADP-ribosylation factor interacting protein 1 (arfaptin 1)	0	0	0	1	0	0	0	1	0	1	1
hsa-miR-152	MIMAT0000438	NM_001025595	ARFIP1	ADP-ribosylation factor interacting protein 1 (arfaptin 1)	0	0	1	1	0	0	0	1	0	1	0
hsa-miR-152	MIMAT0000438	NM_001030007	AP1G1	adaptor-related protein complex 1, gamma 1 subunit	0	0	1	0	0	0	0	1	0	1	1
hsa-miR-152	MIMAT0000438	NM_001031804	MAF	v-maf musculoaponeurotic fibrosarcoma oncogene homolog (avian)	0	0	1	0	0	0	0	1	0	1	1
hsa-miR-152	MIMAT0000438	NM_001039211	ATAD3C	ATPase family, AAA domain containing 3C	0	0	1	1	0	0	0	1	0	1	0
hsa-miR-152	MIMAT0000438	NM_001040101	D4S234E	DNA segment on chromosome 4 (unique) 234 expressed sequence	0	0	1	1	0	0	0	1	0	1	0
hsa-miR-152	MIMAT0000438	NM_001040402	DCUN1D4	DCN1, defective in cullin neddylation 1, domain containing 4 (S. cerevisiae)	0	0	1	1	0	0	0	1	0	1	0
hsa-miR-152	MIMAT0000438	NM_001040424	PRDM15	PR domain containing 15	0	0	1	0	0	0	0	1	0	1	1
hsa-miR-152	MIMAT0000438	NM_001040441	ZBTB8	zinc finger and BTB domain containing 8	0	0	1	0	0	0	0	1	0	1	1
hsa-miR-152	MIMAT0000438	NM_001040446	MTMR12	myotubularin related protein 12	0	0	1	0	0	0	0	1	0	1	1
hsa-miR-152	MIMAT0000438	NM_001040633	PRKAG2	protein kinase, AMP-activated, gamma 2 non-catalytic subunit	0	0	1	0	0	0	0	1	0	1	1
hsa-miR-152	MIMAT0000438	NM_001040712	PTPRD	protein tyrosine phosphatase, receptor type, D	0	0	1	0	0	0	0	1	0	1	1
hsa-miR-152	MIMAT0000438	NM_001042383	CEP63	centrosomal protein 63kDa	0	0	1	1	0	0	0	1	0	1	0
hsa-miR-152	MIMAT0000438	NM_001042400	CEP63	centrosomal protein 63kDa	0	0	1	1	0	0	0	1	0	1	0
hsa-miR-152	MIMAT0000438	NM_001042417	RUFY2	RUN and FYVE domain containing 2	0	0	1	1	0	0	0	1	0	1	0
hsa-miR-152	MIMAT0000438	NM_001042440	CAST	calpastatin	0	0	1	1	0	0	0	1	0	1	0
hsa-miR-152	MIMAT0000438	NM_001042441	CAST	calpastatin	0	0	1	1	0	0	0	1	0	1	0
hsa-miR-152	MIMAT0000438	NM_001042442	CAST	calpastatin	0	0	1	1	0	0	0	1	0	1	0
hsa-miR-152	MIMAT0000438	NM_001042443	CAST	calpastatin	0	0	1	1	0	0	0	1	0	1	0
hsa-miR-152	MIMAT0000438	NM_001042444	CAST	calpastatin	0	0	1	1	0	0	0	1	0	1	0
hsa-miR-152	MIMAT0000438	NM_001042445	CAST	calpastatin	0	0	1	1	0	0	0	1	0	1	0
hsa-miR-152	MIMAT0000438	NM_001042446	CAST	calpastatin	0	0	1	1	0	0	0	1	0	1	0
hsa-miR-152	MIMAT0000438	NM_001044385	ALS2CR4	amyotrophic lateral sclerosis 2 (juvenile) chromosome region, candidate 4	0	0	1	0	0	0	0	1	0	1	1
hsa-miR-152	MIMAT0000438	NM_001066	TNFRSF1B	tumor necrosis factor receptor superfamily, member 1B	0	0	1	1	0	0	0	1	0	1	0
hsa-miR-152	MIMAT0000438	NM_001077700	MIER1	mesoderm induction early response 1 homolog (Xenopus laevis)	0	0	1	1	0	0	0	1	0	1	1
hsa-miR-152	MIMAT0000438	NM_001077701	MIER1	mesoderm induction early response 1 homolog (Xenopus laevis)	0	0	1	1	0	0	0	1	0	1	0
hsa-miR-152	MIMAT0000438	NM_001077702	MIER1	mesoderm induction early response 1 homolog (Xenopus laevis)	0	0	1	1	0	0	0	1	0	1	0

hsa-miR-152	MIMAT0000438	NM_001077703	MIER1	mesoderm induction early response 1 homolog (Xenopus laevis)	0	0	1	1	0	0	0	1	0	1	0
hsa-miR-152	MIMAT0000438	NM_001080114	LDB3	LIM domain binding 3	0	0	1	1	0	0	0	1	0	1	0
hsa-miR-152	MIMAT0000438	NM_001080416	MYBL1	v-myb myeloblastosis viral oncogene homolog (avian)-like 1	0	0	1	0	0	0	0	1	0	1	1
hsa-miR-152	MIMAT0000438	NM_001082577	RBM9	RNA binding motif protein 9	0	0	1	0	0	0	0	1	0	1	1
hsa-miR-152	MIMAT0000438	NM_001105	ACVR1	activin A receptor, type I	0	0	1	1	0	0	1	1	0	1	1
hsa-miR-152	MIMAT0000438	NM_001106	ACVR2B	activin A receptor, type IIB	0	0	1	0	0	0	0	1	0	1	1
hsa-miR-152	MIMAT0000438	NM_001128	AP1G1	adaptor-related protein complex 1, gamma 1 subunit	0	0	1	0	0	0	1	1	0	1	0
hsa-miR-152	MIMAT0000438	NM_001131	CRISP1	cysteine-rich secretory protein 1	0	0	1	1	0	0	0	1	0	1	0
hsa-miR-152	MIMAT0000438	NM_001148	ANK2	ankyrin 2, neuronal	0	0	1	0	0	0	1	1	0	1	1
hsa-miR-152	MIMAT0000438	NM_001286	CLCN6	chloride channel 6	0	0	1	1	0	0	0	1	0	1	0
hsa-miR-152	MIMAT0000438	NM_001304	CPD	carboxypeptidase D	0	0	1	0	0	0	1	1	0	1	1
hsa-miR-152	MIMAT0000438	NM_001358	DHX15	DEAH (Asp-Glu-Ala-His) box polypeptide 15	0	0	0	0	0	0	1	1	0	1	1
hsa-miR-152	MIMAT0000438	NM_001379	DNMT1	DNA (cytosine-5-)-methyltransferase 1	0	0	1	1	0	0	1	1	0	1	1
hsa-miR-152	MIMAT0000438	NM_001390	DTNA	dystrobrevin, alpha	0	0	0	0	0	0	1	1	0	1	1
hsa-miR-152	MIMAT0000438	NM_001400	S1PR1	sphingosine-1-phosphate receptor 1	0	0	1	1	0	0	1	1	0	1	1
hsa-miR-152	MIMAT0000438	NM_001422	ELF5	E74-like factor 5 (ets domain transcription factor)	0	0	1	0	0	0	1	1	0	1	1
hsa-miR-152	MIMAT0000438	NM_001429	EP300	E1A binding protein p300	0	0	0	0	0	0	1	1	0	1	1
hsa-miR-152	MIMAT0000438	NM_001430	EPAS1	endothelial PAS domain protein 1	0	0	1	0	0	0	1	1	0	1	1
hsa-miR-152	MIMAT0000438	NM_001438	ESRRG	estrogen-related receptor gamma	0	0	1	1	0	0	1	1	0	1	1
hsa-miR-152	MIMAT0000438	NM_001543	NDST1	N-deacetylase/N-sulfotransferase (heparan glucosaminyl) 1	0	0	1	0	0	0	1	1	0	1	1
hsa-miR-152	MIMAT0000438	NM_001556	IKBKB	inhibitor of kappa light polypeptide gene enhancer in B-cells, kinase beta	0	0	1	1	0	0	0	1	0	1	0
hsa-miR-152	MIMAT0000438	NM_001560	IL13RA1	interleukin 13 receptor, alpha 1	0	0	0	0	0	0	1	1	0	1	1
hsa-miR-152	MIMAT0000438	NM_001584	MPPED2	metallophosphoesterase domain containing 2	0	0	1	0	0	0	1	1	0	0	1
hsa-miR-152	MIMAT0000438	NM_001650	AQP4	aquaporin 4	0	0	0	0	0	0	1	1	0	1	1
hsa-miR-152	MIMAT0000438	NM_001704	BAI3	brain-specific angiogenesis inhibitor 3	0	0	1	1	0	0	0	1	0	1	0
hsa-miR-152	MIMAT0000438	NM_001746	CANX	calnexin	0	0	1	0	0	0	1	1	0	1	0
hsa-miR-152	MIMAT0000438	NM_001750	CAST	calpastatin	0	0	1	1	0	0	0	1	0	1	0

hsa-miR-152	MIMAT0000438	NM_001844	COL2A1	collagen, type II, alpha 1	0	0	1	1	0	0	1	1	0	1	1
hsa-miR-152	MIMAT0000438	NM_001845	COL4A1	collagen, type IV, alpha 1	0	0	1	1	0	0	0	1	0	1	0
hsa-miR-152	MIMAT0000438	NM_001897	CSPG4	chondroitin sulfate proteoglycan 4	0	0	1	0	0	0	0	1	0	1	1
hsa-miR-152	MIMAT0000438	NM_001924	GADD45A	growth arrest and DNA-damage-inducible, alpha	0	0	1	1	0	0	1	1	0	1	1
hsa-miR-152	MIMAT0000438	NM_001935	DPP4	dipeptidyl-peptidase 4 (CD26, adenosine deaminase complexing protein 2)	0	0	0	1	0	0	1	1	0	1	1
hsa-miR-152	MIMAT0000438	NM_001949	E2F3	E2F transcription factor 3	0	0	1	0	0	0	1	1	0	1	1
hsa-miR-152	MIMAT0000438	NM_001974	EMR1	egf-like module containing, mucin-like, hormone receptor-like 1	0	0	1	1	0	0	0	1	0	1	0
hsa-miR-152	MIMAT0000438	NM_001981	EPS15	epidermal growth factor receptor pathway substrate 15	0	0	1	0	0	0	0	1	0	1	1
hsa-miR-152	MIMAT0000438	NM_001982	ERBB3	v-erb-b2 erythroblastic leukemia viral oncogene homolog 3 (avian)	0	0	1	1	0	0	0	1	0	1	1
hsa-miR-152	MIMAT0000438	NM_002019	FLT1	fms-related tyrosine kinase 1 (vascular endothelial growth factor/vascular permeability factor receptor)	0	0	1	1	0	0	0	1	0	1	0
hsa-miR-152	MIMAT0000438	NM_002024	FMR1	fragile X mental retardation 1	0	0	1	1	0	0	0	1	0	1	1
hsa-miR-152	MIMAT0000438	NM_002045	GAP43	growth associated protein 43	0	0	1	0	0	0	1	1	0	1	1
hsa-miR-152	MIMAT0000438	NM_002076	GNS	glucosamine (N-acetyl)-6-sulfatase (Sanfilippo disease IIID)	0	0	1	1	0	0	0	1	0	1	0
hsa-miR-152	MIMAT0000438	NM_002193	INHBB	inhibin, beta B	0	0	1	1	0	0	1	1	0	1	1
hsa-miR-152	MIMAT0000438	NM_002205	ITGA5	integrin, alpha 5 (fibronectin receptor, alpha polypeptide)	0	0	1	1	0	0	1	1	0	1	1
hsa-miR-152	MIMAT0000438	NM_002207	ITGA9	integrin, alpha 9	0	0	0	0	0	0	1	1	0	1	1
hsa-miR-152	MIMAT0000438	NM_002214	ITGB8	integrin, beta 8	0	0	1	0	0	0	1	1	0	1	1
hsa-miR-152	MIMAT0000438	NM_002265	KPNB1	karyopherin (importin) beta 1	0	0	0	0	0	0	1	1	0	1	1
hsa-miR-152	MIMAT0000438	NM_002296	LBR	lamin B receptor	0	0	0	1	0	0	1	1	0	1	1
hsa-miR-152	MIMAT0000438	NM_002359	MAFG	v-maf musculoaponeurotic fibrosarcoma oncogene homolog G (avian)	0	0	1	1	0	0	0	1	0	1	0
hsa-miR-152	MIMAT0000438	NM_002427	MMP13	matrix metallopeptidase 13 (collagenase 3)	0	0	1	1	0	0	0	1	0	1	0
hsa-miR-152	MIMAT0000438	NM_002428	MMP15	matrix metallopeptidase 15 (membrane-inserted)	0	0	1	1	0	0	0	1	0	1	1
hsa-miR-152	MIMAT0000438	NM_002480	PPP1R12A	protein phosphatase 1, regulatory (inhibitor) subunit 12A	0	0	1	1	0	0	1	1	0	1	1
hsa-miR-152	MIMAT0000438	NM_002500	NEUROD1	neurogenic differentiation 1	0	0	1	1	0	0	0	1	0	1	0
hsa-miR-152	MIMAT0000438	NM_002515	NOVA1	neuro-oncological ventral antigen 1	0	0	0	0	0	0	1	1	0	1	1
hsa-miR-152	MIMAT0000438	NM_002522	NPTX1	neuronal pentraxin I	0	0	1	1	0	0	0	1	0	1	1
hsa-miR-152	MIMAT0000438	NM_002523	NPTX2	neuronal pentraxin II	0	0	1	1	0	0	0	1	0	1	0

hsa-miR-152	MIMAT0000438	NM_002612	PDK4	pyruvate dehydrogenase kinase, isozyme 4	0	0	1	0	0	0	1	1	0	1	1
hsa-miR-152	MIMAT0000438	NM_002641	PIGA	phosphatidylinositol glycan anchor biosynthesis, class A (paroxysmal nocturnal hemoglobinuria)	0	0	1	1	0	0	0	1	0	1	0
hsa-miR-152	MIMAT0000438	NM_002709	PPP1CB	protein phosphatase 1, catalytic subunit, beta isoform	0	0	1	0	0	0	1	1	0	1	1
hsa-miR-152	MIMAT0000438	NM_002714	PPP1R10	protein phosphatase 1, regulatory (inhibitor) subunit 10	0	0	1	1	0	0	1	0	0	1	1
hsa-miR-152	MIMAT0000438	NM_002845	PTPRM	protein tyrosine phosphatase, receptor type, M	0	0	0	0	0	0	1	1	0	1	1
hsa-miR-152	MIMAT0000438	NM_002855	PVRL1	poliovirus receptor-related 1 (herpesvirus entry mediator C)	0	0	1	0	0	0	0	1	0	1	1
hsa-miR-152	MIMAT0000438	NM_002858	ABCD3	ATP-binding cassette, sub-family D (ALD), member 3	0	0	1	1	0	0	0	1	0	1	0
hsa-miR-152	MIMAT0000438	NM_002941	ROBO1	roundabout, axon guidance receptor, homolog 1 (Drosophila)	0	0	1	1	0	0	1	1	0	1	1
hsa-miR-152	MIMAT0000438	NM_002942	ROBO2	roundabout, axon guidance receptor, homolog 2 (Drosophila)	0	0	0	1	0	0	0	1	0	1	1
hsa-miR-152	MIMAT0000438	NM_002957	RXRA	retinoid X receptor, alpha	0	0	1	0	0	0	0	1	0	1	1
hsa-miR-152	MIMAT0000438	NM_003076	SMARCD1	SWI/SNF related, matrix associated, actin dependent regulator of chromatin, subfamily d, member 1	0	0	1	0	0	0	1	1	0	1	1
hsa-miR-152	MIMAT0000438	NM_003108	SOX11	SRY (sex determining region Y)-box 11	0	0	1	0	0	0	0	1	0	1	1
hsa-miR-152	MIMAT0000438	NM_003144	SSR1	signal sequence receptor, alpha (translocon-associated protein alpha)	0	0	0	1	0	0	1	1	0	1	1
hsa-miR-152	MIMAT0000438	NM_003185	TAF4	TAF4 RNA polymerase II, TATA box binding protein (TBP)-associated factor, 135kDa	0	0	1	0	0	0	1	1	0	1	1
hsa-miR-152	MIMAT0000438	NM_003234	TFRC	transferrin receptor (p90, CD71)	0	0	1	0	0	0	1	1	0	1	1
hsa-miR-152	MIMAT0000438	NM_003236	TGFA	transforming growth factor, alpha	0	0	1	1	0	0	1	1	0	1	1
hsa-miR-152	MIMAT0000438	NM_003299	HSP90B1	heat shock protein 90kDa beta (Grp94), member 1	0	0	0	0	0	0	1	1	0	1	1
hsa-miR-152	MIMAT0000438	NM_003338	UBE2D1	ubiquitin-conjugating enzyme E2D 1 (UBC4/5 homolog, yeast)	0	0	1	1	0	0	1	1	0	1	1
hsa-miR-152	MIMAT0000438	NM_003356	UCP3	uncoupling protein 3 (mitochondrial, proton carrier)	0	0	0	0	0	0	1	1	0	1	1
hsa-miR-152	MIMAT0000438	NM_003363	USP4	ubiquitin specific peptidase 4 (proto-oncogene)	0	0	1	1	0	0	0	1	0	1	0
hsa-miR-152	MIMAT0000438	NM_003394	WNT10B	wingless-type MMTV integration site family, member 10B	0	0	0	1	0	0	1	1	0	1	1
hsa-miR-152	MIMAT0000438	NM_003404	YWHAB	tyrosine 3-monooxygenase/tryptophan 5-monooxygenase activation protein, beta polypeptide	0	0	1	1	0	0	1	1	0	1	1
hsa-miR-152	MIMAT0000438	NM_003478	CUL5	cullin 5	0	0	1	1	0	0	1	1	0	1	1
hsa-miR-152	MIMAT0000438	NM_003488	AKAP1	A kinase (PRKA) anchor protein 1	0	0	1	1	0	0	1	1	0	1	1
hsa-miR-152	MIMAT0000438	NM_003492	TMEM187	transmembrane protein 187	0	0	1	1	0	0	0	1	0	1	0
hsa-miR-152	MIMAT0000438	NM_003498	SNN	stannin	0	0	1	0	0	0	1	1	0	1	1
hsa-miR-152	MIMAT0000438	NM_003506	FZD6	frizzled homolog 6 (Drosophila)	0	0	1	1	0	0	0	1	0	1	0

hsa-miR-152	MIMAT0000438	NM_003583	DYRK2	dual-specificity tyrosine-(Y)-phosphorylation regulated kinase 2	0	0	1	1	0	0	1	1	0	1	0
hsa-miR-152	MIMAT0000438	NM_003625	PPFIA2	protein tyrosine phosphatase, receptor type, f polypeptide (PTPRF), interacting protein (liprin), alpha 2	0	0	0	0	0	0	1	1	0	1	1
hsa-miR-152	MIMAT0000438	NM_003629	PIK3R3	phosphoinositide-3-kinase, regulatory subunit 3 (gamma)	0	0	1	0	0	0	1	1	0	1	1
hsa-miR-152	MIMAT0000438	NM_003672	CDC14A	CDC14 cell division cycle 14 homolog A (S. cerevisiae)	0	0	1	1	0	0	1	1	0	1	0
hsa-miR-152	MIMAT0000438	NM_003718	CDC2L5	cell division cycle 2-like 5 (cholinesterase-related cell division controller)	0	0	0	0	0	0	1	1	0	1	1
hsa-miR-152	MIMAT0000438	NM_003762	VAMP4	vesicle-associated membrane protein 4	0	0	1	1	0	0	0	1	0	1	0
hsa-miR-152	MIMAT0000438	NM_003787	NOL4	nucleolar protein 4	0	0	1	0	0	0	0	1	0	1	1
hsa-miR-152	MIMAT0000438	NM_003828	MTMR1	myotubularin related protein 1	0	0	1	1	0	0	0	1	0	1	0
hsa-miR-152	MIMAT0000438	NM_003829	MPDZ	multiple PDZ domain protein	0	0	1	1	0	0	0	1	0	1	0
hsa-miR-152	MIMAT0000438	NM_003861	WDR22	WD repeat domain 22	0	0	1	0	0	0	0	1	0	1	1
hsa-miR-152	MIMAT0000438	NM_003873	NRP1	neuropilin 1	0	0	1	1	0	0	1	1	0	1	1
hsa-miR-152	MIMAT0000438	NM_003885	CDK5R1	cyclin-dependent kinase 5, regulatory subunit 1 (p35)	0	0	1	1	0	0	1	1	0	1	1
hsa-miR-152	MIMAT0000438	NM_003895	SYNJ1	synaptojanin 1	0	0	0	1	0	0	1	1	0	1	1
hsa-miR-152	MIMAT0000438	NM_003941	WASL	Wiskott-Aldrich syndrome-like	0	0	1	1	0	0	0	1	0	1	0
hsa-miR-152	MIMAT0000438	NM_003972	BTAF1	BTAF1 RNA polymerase II, B-TFIID transcription factor-associated, 170kDa (Mot1 homolog, S. cerevisiae)	0	0	0	1	0	0	1	1	0	1	1
hsa-miR-152	MIMAT0000438	NM_004050	BCL2L2	BCL2-like 2	0	0	0	0	0	0	1	1	0	1	1
hsa-miR-152	MIMAT0000438	NM_004064	CDKN1B	cyclin-dependent kinase inhibitor 1B (p27, Kip1)	0	0	0	0	0	0	1	1	0	1	1
hsa-miR-152	MIMAT0000438	NM_004077	CS	citrate synthase	0	0	0	0	0	0	1	1	0	1	1
hsa-miR-152	MIMAT0000438	NM_004093	EFNB2	ephrin-B2	0	0	1	0	0	0	1	1	0	1	1
hsa-miR-152	MIMAT0000438	NM_004098	EMX2	empty spiracles homeobox 2	0	0	1	0	0	0	1	1	0	1	1
hsa-miR-152	MIMAT0000438	NM_004124	GMFB	glia maturation factor, beta	0	0	1	1	0	0	0	1	0	1	1
hsa-miR-152	MIMAT0000438	NM_004177	STX3	syntaxin 3	0	0	1	0	0	0	1	1	0	1	1
hsa-miR-152	MIMAT0000438	NM_004227	PSCD3	pleckstrin homology, Sec7 and coiled-coil domains 3	0	0	1	0	0	0	1	1	0	1	1
hsa-miR-152	MIMAT0000438	NM_004235	KLF4	Kruppel-like factor 4 (gut)	0	0	1	0	0	0	1	1	0	1	1
hsa-miR-152	MIMAT0000438	NM_004253	PLAA	phospholipase A2-activating protein	0	0	0	0	0	0	1	1	0	1	1
hsa-miR-152	MIMAT0000438	NM_004261	SELENOF	15 kDa selenoprotein	0	0	1	1	0	0	0	1	0	1	0
hsa-miR-152	MIMAT0000438	NM_004272	HOMER1	homer homolog 1 (Drosophila)	0	0	1	1	0	0	0	1	0	1	0

hsa-miR-152	MIMAT0000438	NM_004299	ABCB7	ATP-binding cassette, sub-family B (MDR/TAP), member 7	0	0	1	1	0	0	1	1	0	1	1
hsa-miR-152	MIMAT0000438	NM_004358	CDC25B	cell division cycle 25 homolog B (S. pombe)	0	0	0	0	0	0	1	1	0	1	1
hsa-miR-152	MIMAT0000438	NM_004397	DDX6	DEAD (Asp-Glu-Ala-Asp) box polypeptide 6	0	0	1	1	0	0	0	1	0	1	1
hsa-miR-152	MIMAT0000438	NM_004417	DUSP1	dual specificity phosphatase 1	0	0	0	0	0	0	1	1	0	1	1
hsa-miR-152	MIMAT0000438	NM_004432	ELAVL2	ELAV (embryonic lethal, abnormal vision, Drosophila)-like 2 (Hu antigen B)	0	0	1	0	0	0	0	1	0	1	1
hsa-miR-152	MIMAT0000438	NM_004458	ACSL4	acyl-CoA synthetase long-chain family member 4	0	0	0	0	0	0	1	1	0	1	1
hsa-miR-152	MIMAT0000438	NM_004504	HRB	HIV-1 Rev binding protein	0	0	1	0	0	0	1	1	0	1	1
hsa-miR-152	MIMAT0000438	NM_004505	USP6	ubiquitin specific peptidase 6 (Tre-2 oncogene)	0	0	1	0	0	0	1	1	0	1	1
hsa-miR-152	MIMAT0000438	NM_004514	FOXK2	forkhead box K2	0	0	1	0	0	0	1	1	0	1	1
hsa-miR-152	MIMAT0000438	NM_004525	LRP2	low density lipoprotein-related protein 2	0	0	1	1	0	0	0	1	0	1	0
hsa-miR-152	MIMAT0000438	NM_004575	POU4F2	POU class 4 homeobox 2	0	0	1	0	0	0	1	1	0	1	1
hsa-miR-152	MIMAT0000438	NM_004595	SMS	spermine synthase	0	0	1	1	0	0	0	1	0	1	1
hsa-miR-152	MIMAT0000438	NM_004631	LRP8	low density lipoprotein receptor-related protein 8, apolipoprotein e receptor	0	0	1	0	0	0	1	1	0	1	0
hsa-miR-152	MIMAT0000438	NM_004634	BRPF1	bromodomain and PHD finger containing, 1	0	0	1	0	0	0	1	1	0	1	0
hsa-miR-152	MIMAT0000438	NM_004641	MLLT10	myeloid/lymphoid or mixed-lineage leukemia (trithorax homolog, Drosophila); translocated to, 10	0	0	0	1	0	0	1	1	0	1	0
hsa-miR-152	MIMAT0000438	NM_004670	PAPSS2	3''-phosphoadenosine 5''-phosphosulfate synthase 2	0	0	1	1	0	0	0	1	0	1	0
hsa-miR-152	MIMAT0000438	NM_004714	DYRK1B	dual-specificity tyrosine-(Y)-phosphorylation regulated kinase 1B	0	0	0	0	0	0	1	1	0	1	1
hsa-miR-152	MIMAT0000438	NM_004719	SFRS2IP	splicing factor, arginine/serine-rich 2, interacting protein	0	0	1	1	0	0	1	1	0	1	1
hsa-miR-152	MIMAT0000438	NM_004742	MAGI1	membrane associated guanylate kinase, WW and PDZ domain containing 1	0	0	1	1	0	0	0	1	0	1	0
hsa-miR-152	MIMAT0000438	NM_004755	RPS6KA5	ribosomal protein S6 kinase, 90kDa, polypeptide 5	0	0	0	1	0	0	1	1	0	1	1
hsa-miR-152	MIMAT0000438	NM_004758	BZRAP1	benzodiazapine receptor (peripheral) associated protein 1	0	0	1	0	0	0	1	1	0	1	1
hsa-miR-152	MIMAT0000438	NM_004768	SFRS11	splicing factor, arginine/serine-rich 11	0	0	1	1	0	0	1	1	0	1	1
hsa-miR-152	MIMAT0000438	NM_004776	B4GALT5	UDP-Gal:betaGlcNAc beta 1,4- galactosyltransferase, polypeptide 5	0	0	1	1	0	0	1	1	0	1	1
hsa-miR-152	MIMAT0000438	NM_004842	AKAP7	A kinase (PRKA) anchor protein 7	0	0	0	0	0	0	1	1	0	1	1
hsa-miR-152	MIMAT0000438	NM_004898	CLOCK	clock homolog (mouse)	0	0	0	0	0	0	1	1	0	1	1
hsa-miR-152	MIMAT0000438	NM_004947	DOCK3	dedicator of cytokinesis 3	0	0	0	0	0	0	1	1	0	1	1
hsa-miR-152	MIMAT0000438	NM_004973	JARID2	jumonji, AT rich interactive domain 2	0	0	1	0	0	0	1	1	0	1	0

hsa-miR-152	MIMAT0000438	NM_004980	KCND3	potassium voltage-gated channel, Shal-related subfamily, member 3	0	0	0	0	0	0	1	1	0	1	1
hsa-miR-152	MIMAT0000438	NM_004992	MECP2	methyl CpG binding protein 2 (Rett syndrome)	0	0	0	0	0	0	1	1	0	1	1
hsa-miR-152	MIMAT0000438	NM_005102	FEZ2	fasciculation and elongation protein zeta 2 (zygin II)	0	0	1	1	0	0	0	1	0	1	0
hsa-miR-152	MIMAT0000438	NM_005109	OXSR1	oxidative-stress responsive 1	0	0	1	0	0	0	1	1	0	1	1
hsa-miR-152	MIMAT0000438	NM_005137	DGCR2	DiGeorge syndrome critical region gene 2	0	0	1	1	0	0	0	1	0	1	0
hsa-miR-152	MIMAT0000438	NM_005147	DNAJA3	DnaJ (Hsp40) homolog, subfamily A, member 3	0	0	0	0	0	0	1	1	0	1	1
hsa-miR-152	MIMAT0000438	NM_005238	ETS1	v-ets erythroblastosis virus E26 oncogene homolog 1 (avian)	0	0	1	1	0	0	0	1	0	1	0
hsa-miR-152	MIMAT0000438	NM_005277	GPM6A	glycoprotein M6A	0	0	1	1	0	0	1	1	0	1	1
hsa-miR-152	MIMAT0000438	NM_005313	PDIA3	protein disulfide isomerase family A, member 3	0	0	0	1	0	0	1	1	0	1	1
hsa-miR-152	MIMAT0000438	NM_005316	GTF2H1	general transcription factor IIH, polypeptide 1, 62kDa	0	0	0	1	0	0	1	1	0	1	1
hsa-miR-152	MIMAT0000438	NM_005329	HAS3	hyaluronan synthase 3	0	0	0	0	0	0	1	1	0	1	1
hsa-miR-152	MIMAT0000438	NM_005342	HMGB3	high-mobility group box 3	0	0	1	0	0	0	1	1	0	1	1
hsa-miR-152	MIMAT0000438	NM_005406	ROCK1	Rho-associated, coiled-coil containing protein kinase 1	0	0	1	0	0	0	0	1	0	1	1
hsa-miR-152	MIMAT0000438	NM_005430	WNT1	wingless-type MMTV integration site family, member 1	0	0	1	1	0	0	1	1	0	1	1
hsa-miR-152	MIMAT0000438	NM_005436	CCDC6	coiled-coil domain containing 6	0	0	1	1	0	0	0	1	0	1	0
hsa-miR-152	MIMAT0000438	NM_005450	NOG	noggin	0	0	1	1	0	0	1	1	0	1	1
hsa-miR-152	MIMAT0000438	NM_005461	MAFB	v-maf musculoaponeurotic fibrosarcoma oncogene homolog B (avian)	0	0	1	1	0	0	1	1	0	1	1
hsa-miR-152	MIMAT0000438	NM_005502	ABCA1	ATP-binding cassette, sub-family A (ABC1), member 1	0	0	1	1	0	0	1	1	0	1	1
hsa-miR-152	MIMAT0000438	NM_005509	DMXL1	Dmx-like 1	0	0	1	1	0	0	1	1	0	1	1
hsa-miR-152	MIMAT0000438	NM_005544	IRS1	insulin receptor substrate 1	0	0	1	1	0	0	0	1	0	1	0
hsa-miR-152	MIMAT0000438	NM_005604	POU3F2	POU class 3 homeobox 2	0	0	0	0	0	0	1	1	0	1	1
hsa-miR-152	MIMAT0000438	NM_005639	SYT1	synaptotagmin I	0	0	1	0	0	0	1	1	0	1	1
hsa-miR-152	MIMAT0000438	NM_005652	TERF2	telomeric repeat binding factor 2	0	0	1	1	0	0	0	1	0	1	0
hsa-miR-152	MIMAT0000438	NM_005700	DPP3	dipeptidyl-peptidase 3	0	0	1	0	0	0	1	1	0	1	1
hsa-miR-152	MIMAT0000438	NM_005765	ATP6AP2	ATPase, H+ transporting, lysosomal accessory protein 2	0	0	0	1	0	0	1	1	0	1	1
hsa-miR-152	MIMAT0000438	NM_005840	SPRY3	sprouty homolog 3 (Drosophila)	0	0	1	0	0	0	1	0	0	1	1
hsa-miR-152	MIMAT0000438	NM_005924	MEOX2	mesenchyme homeobox 2	0	0	0	1	0	0	1	1	0	1	1

hsa-miR-152	MIMAT0000438	NM_005933	MLL	myeloid/lymphoid or mixed-lineage leukemia (trithorax homolog, Drosophila)	0	0	1	1	0	0	0	1	0	1	1
hsa-miR-152	MIMAT0000438	NM_005937	MLLT6	myeloid/lymphoid or mixed-lineage leukemia (trithorax homolog, Drosophila); translocated to, 6	0	0	1	0	0	0	0	1	0	1	1
hsa-miR-152	MIMAT0000438	NM_005955	MTF1	metal-regulatory transcription factor 1	0	0	1	1	0	0	1	1	0	1	1
hsa-miR-152	MIMAT0000438	NM_006029	PNMA1	paraneoplastic antigen MA1	0	0	1	1	0	0	0	1	0	1	0
hsa-miR-152	MIMAT0000438	NM_006035	CDC42BPB	CDC42 binding protein kinase beta (DMPK-like)	0	0	0	0	0	0	1	1	0	1	1
hsa-miR-152	MIMAT0000438	NM_006054	RTN3	reticulon 3	0	0	1	1	0	0	0	1	0	1	0
hsa-miR-152	MIMAT0000438	NM_006134	TMEM50B	transmembrane protein 50B	0	0	1	1	0	0	0	1	0	1	0
hsa-miR-152	MIMAT0000438	NM_006251	PRKAA1	protein kinase, AMP-activated, alpha 1 catalytic subunit	0	0	1	0	0	0	1	1	0	1	1
hsa-miR-152	MIMAT0000438	NM_006277	ITSN2	intersectin 2	0	0	1	1	0	0	1	1	0	1	1
hsa-miR-152	MIMAT0000438	NM_006327	TIMM23	translocase of inner mitochondrial membrane 23 homolog (yeast)	0	0	1	1	0	0	0	1	0	1	0
hsa-miR-152	MIMAT0000438	NM_006352	ZNF238	zinc finger protein 238	0	0	1	1	0	0	1	1	0	1	1
hsa-miR-152	MIMAT0000438	NM_006369	LRRC41	leucine rich repeat containing 41	0	0	1	0	0	0	1	1	0	1	1
hsa-miR-152	MIMAT0000438	NM_006390	IPO8	importin 8	0	0	1	1	0	0	0	1	0	1	0
hsa-miR-152	MIMAT0000438	NM_006421	ARFGEF1	ADP-ribosylation factor guanine nucleotide-exchange factor 1(brefeldin A-inhibited)	0	0	1	0	0	0	1	1	0	1	1
hsa-miR-152	MIMAT0000438	NM_006459	ERLIN1	ER lipid raft associated 1	0	0	1	1	0	0	1	1	0	1	1
hsa-miR-152	MIMAT0000438	NM_006464	TGOLN2	trans-golgi network protein 2	0	0	1	1	0	0	0	1	0	1	0
hsa-miR-152	MIMAT0000438	NM_006472	TXNIP	thioredoxin interacting protein	0	0	0	0	0	0	1	1	0	1	1
hsa-miR-152	MIMAT0000438	NM_006482	DYRK2	dual-specificity tyrosine-(Y)-phosphorylation regulated kinase 2	0	0	1	1	0	0	1	1	0	1	0
hsa-miR-152	MIMAT0000438	NM_006484	DYRK1B	dual-specificity tyrosine-(Y)-phosphorylation regulated kinase 1B	0	0	1	0	0	0	1	1	0	1	0
hsa-miR-152	MIMAT0000438	NM_006516	SLC2A1	solute carrier family 2 (facilitated glucose transporter), member 1	0	0	1	1	0	0	1	1	0	1	1
hsa-miR-152	MIMAT0000438	NM_006526	ZNF217	zinc finger protein 217	0	0	0	0	0	0	1	1	0	1	1
hsa-miR-152	MIMAT0000438	NM_006538	BCL2L11	BCL2-like 11 (apoptosis facilitator)	0	0	1	1	0	0	0	1	0	1	1
hsa-miR-152	MIMAT0000438	NM_006581	FUT9	fucosyltransferase 9 (alpha (1,3) fucosyltransferase)	0	0	1	1	0	0	0	1	0	1	0
hsa-miR-152	MIMAT0000438	NM_006599	NFAT5	nuclear factor of activated T-cells 5, tonicity-responsive	0	0	1	0	0	0	1	1	0	1	1
hsa-miR-152	MIMAT0000438	NM_006628	ARPP-19	cyclic AMP phosphoprotein, 19 kD	0	0	1	1	0	0	1	1	0	1	1
hsa-miR-152	MIMAT0000438	NM_006698	BLCAP	bladder cancer associated protein	0	0	0	0	0	0	1	1	0	1	1
hsa-miR-152	MIMAT0000438	NM_006702	PNPLA6	patatin-like phospholipase domain containing 6	0	0	0	1	0	0	1	1	0	1	1

hsa-miR-152	MIMAT0000438	NM_006722	MITF	microphthalmia-associated transcription factor	0	0	1	1	0	0	1	1	0	1	0
hsa-miR-152	MIMAT0000438	NM_006732	FOSB	FBJ murine osteosarcoma viral oncogene homolog B	0	0	0	0	0	0	1	1	0	1	1
hsa-miR-152	MIMAT0000438	NM_006775	QKI	quaking homolog, KH domain RNA binding (mouse)	0	0	1	1	0	0	0	1	0	1	0
hsa-miR-152	MIMAT0000438	NM_006788	RALBP1	ralA binding protein 1	0	0	1	0	0	0	1	1	0	1	1
hsa-miR-152	MIMAT0000438	NM_006851	GLIPR1	GLI pathogenesis-related 1 (glioma)	0	0	1	1	0	0	0	1	0	1	0
hsa-miR-152	MIMAT0000438	NM_006861	RAB35	RAB35, member RAS oncogene family	0	0	1	0	0	0	1	1	0	1	1
hsa-miR-152	MIMAT0000438	NM_006885	ZFHX3	zinc finger homeobox 3	0	0	1	0	0	0	0	1	0	1	1
hsa-miR-152	MIMAT0000438	NM_006914	RORB	RAR-related orphan receptor B	0	0	1	0	0	0	1	1	0	1	1
hsa-miR-152	MIMAT0000438	NM_006930	SKP1	S-phase kinase-associated protein 1	0	0	1	1	0	0	1	1	0	1	0
hsa-miR-152	MIMAT0000438	NM_006938	SNRPD1	small nuclear ribonucleoprotein D1 polypeptide 16kDa	0	0	1	1	0	0	0	1	0	1	0
hsa-miR-152	MIMAT0000438	NM_006940	SOX5	SRY (sex determining region Y)-box 5	0	0	0	0	0	0	1	1	0	1	1
hsa-miR-152	MIMAT0000438	NM_007008	RTN4	reticulon 4	0	0	1	1	0	0	0	1	0	1	0
hsa-miR-152	MIMAT0000438	NM_007038	ADAMTS5	ADAM metallopeptidase with thrombospondin type 1 motif, 5 (aggrecanase-2)	0	0	1	0	0	0	0	1	0	1	1
hsa-miR-152	MIMAT0000438	NM_007067	MYST2	MYST histone acetyltransferase 2	0	0	1	0	0	0	0	1	0	1	1
hsa-miR-152	MIMAT0000438	NM_007078	LDB3	LIM domain binding 3	0	0	1	1	0	0	0	1	0	1	0
hsa-miR-152	MIMAT0000438	NM_007174	CIT	citron (rho-interacting, serine/threonine kinase 21)	0	0	0	0	0	0	1	1	0	1	1
hsa-miR-152	MIMAT0000438	NM_007347	AP4E1	adaptor-related protein complex 4, epsilon 1 subunit	0	0	1	0	0	0	0	1	0	1	1
hsa-miR-152	MIMAT0000438	NM_007375	TARDBP	TAR DNA binding protein	0	0	1	0	0	0	1	1	0	1	0
hsa-miR-152	MIMAT0000438	NM_012082	ZFPM2	zinc finger protein, multitype 2	0	0	0	0	0	0	1	1	0	1	1
hsa-miR-152	MIMAT0000438	NM_012137	DDAH1	dimethylarginine dimethylaminohydrolase 1	0	0	1	1	0	0	0	1	0	1	0
hsa-miR-152	MIMAT0000438	NM_012199	EIF2C1	eukaryotic translation initiation factor 2C, 1	0	0	1	1	0	0	1	1	0	1	1
hsa-miR-152	MIMAT0000438	NM_012214	MGAT4A	mannosyl (alpha-1,3-)-glycoprotein beta-1,4-N-acetylglucosaminyltransferase, isozyme A	0	0	0	1	0	0	0	1	0	1	1
hsa-miR-152	MIMAT0000438	NM_012219	MRAS	muscle RAS oncogene homolog	0	0	1	1	0	0	0	1	0	1	0
hsa-miR-152	MIMAT0000438	NM_012317	LDOC1	leucine zipper, down-regulated in cancer 1	0	0	1	1	0	0	0	1	0	1	0
hsa-miR-152	MIMAT0000438	NM_012323	MAFF	v-maf musculoaponeurotic fibrosarcoma oncogene homolog F (avian)	0	0	1	1	0	0	0	1	0	1	0
hsa-miR-152	MIMAT0000438	NM_012329	MMD	monocyte to macrophage differentiation-associated	0	0	1	1	0	0	1	1	0	1	1
hsa-miR-152	MIMAT0000438	NM_012393	PFAS	phosphoribosylformylglycinamidine synthase (FGAR amidotransferase)	0	0	1	1	0	0	0	1	0	1	0

hsa-miR-152	MIMAT0000438	NM_012395	PFTK1	PFTAIRE protein kinase 1	0	0	1	1	0	0	0	1	0	1	0
hsa-miR-152	MIMAT0000438	NM_012428	NPTN	neuroplastin	0	0	0	1	0	0	1	1	0	1	1
hsa-miR-152	MIMAT0000438	NM_012437	SNAPIN	SNAP-associated protein	0	0	1	1	0	0	0	1	0	1	0
hsa-miR-152	MIMAT0000438	NM_012470	TNPO3	transportin 3	0	0	1	0	0	0	1	0	0	1	1
hsa-miR-152	MIMAT0000438	NM_013261	PPARGC1A	peroxisome proliferator-activated receptor gamma, coactivator 1 alpha	0	0	1	0	0	0	1	1	0	1	1
hsa-miR-152	MIMAT0000438	NM_013330	NME7	non-metastatic cells 7, protein expressed in (nucleoside-diphosphate kinase)	0	0	0	0	0	0	1	1	0	1	1
hsa-miR-152	MIMAT0000438	NM_013410	AK3L1	adenylate kinase 3-like 1	0	0	1	1	0	0	0	1	0	1	0
hsa-miR-152	MIMAT0000438	NM_013411	AK2	adenylate kinase 2	0	0	1	1	0	0	0	1	0	1	0
hsa-miR-152	MIMAT0000438	NM_013449	BAZ2A	bromodomain adjacent to zinc finger domain, 2A	0	0	1	0	0	0	1	1	0	1	1
hsa-miR-152	MIMAT0000438	NM_013450	BAZ2B	bromodomain adjacent to zinc finger domain, 2B	0	0	1	0	0	0	0	1	0	1	1
hsa-miR-152	MIMAT0000438	NM_014112	TRPS1	trichorhinophalangeal syndrome I	0	0	1	0	0	0	1	1	0	1	1
hsa-miR-152	MIMAT0000438	NM_014216	ITPK1	inositol 1,3,4-triphosphate 5/6 kinase	0	0	0	0	0	0	1	1	0	1	1
hsa-miR-152	MIMAT0000438	NM_014246	CELSR1	cadherin, EGF LAG seven-pass G-type receptor 1 (flamingo homolog, Drosophila)	0	0	1	1	0	0	0	1	0	1	0
hsa-miR-152	MIMAT0000438	NM_014278	HSPA4L	heat shock 70kDa protein 4-like	0	0	0	0	0	0	1	1	0	1	1
hsa-miR-152	MIMAT0000438	NM_014331	SLC7A11	solute carrier family 7, (cationic amino acid transporter, y+ system) member 11	0	0	1	0	0	0	0	1	0	1	1
hsa-miR-152	MIMAT0000438	NM_014392	D4S234E	DNA segment on chromosome 4 (unique) 234 expressed sequence	0	0	1	1	0	0	0	1	0	1	0
hsa-miR-152	MIMAT0000438	NM_014417	BBC3	BCL2 binding component 3	0	0	1	0	0	0	1	1	0	1	0
hsa-miR-152	MIMAT0000438	NM_014494	TNRC6A	trinucleotide repeat containing 6A	0	0	0	1	0	0	1	1	0	1	1
hsa-miR-152	MIMAT0000438	NM_014562	OTX1	orthodenticle homeobox 1	0	0	1	0	0	0	1	1	0	1	1
hsa-miR-152	MIMAT0000438	NM_014655	SLC25A44	solute carrier family 25, member 44	0	0	1	0	0	0	1	1	0	1	1
hsa-miR-152	MIMAT0000438	NM_014682	ST18	suppression of tumorigenicity 18 (breast carcinoma) (zinc finger protein)	0	0	1	1	0	0	1	1	0	1	0
hsa-miR-152	MIMAT0000438	NM_014721	PHACTR2	phosphatase and actin regulator 2	0	0	1	1	0	0	0	1	0	1	1
hsa-miR-152	MIMAT0000438	NM_014723	SNPH	syntaphilin	0	0	1	0	0	0	1	1	0	1	1
hsa-miR-152	MIMAT0000438	NM_014730	KIAA0152	KIAA0152	0	0	1	0	0	0	1	1	0	1	1
hsa-miR-152	MIMAT0000438	NM_014731	ProSAPiP1	ProSAPiP1 protein	0	0	1	0	0	0	0	1	0	1	1
hsa-miR-152	MIMAT0000438	NM_014743	KIAA0232	KIAA0232	0	0	1	1	0	0	0	1	0	1	1
hsa-miR-152	MIMAT0000438	NM_014786	ARHGEF17	Rho guanine nucleotide exchange factor (GEF) 17	0	0	1	0	0	0	1	1	0	1	1

hsa-miR-152	MIMAT0000438	NM_014800	ELMO1	engulfment and cell motility 1	0	0	1	0	0	0	1	1	0	1	0
hsa-miR-152	MIMAT0000438	NM_014810	CEP350	centrosomal protein 350kDa	0	0	1	0	0	0	1	1	0	1	1
hsa-miR-152	MIMAT0000438	NM_014820	TOMM70A	translocase of outer mitochondrial membrane 70 homolog A (S. cerevisiae)	0	0	1	0	0	0	1	1	0	1	1
hsa-miR-152	MIMAT0000438	NM_014838	ZBED4	zinc finger, BED-type containing 4	0	0	1	0	0	0	0	1	0	1	1
hsa-miR-152	MIMAT0000438	NM_014841	SNAP91	synaptosomal-associated protein, 91kDa homolog (mouse)	0	0	1	1	0	0	0	1	0	1	0
hsa-miR-152	MIMAT0000438	NM_014901	RNF44	ring finger protein 44	0	0	1	0	0	0	1	1	0	1	1
hsa-miR-152	MIMAT0000438	NM_014916	LMTK2	lemur tyrosine kinase 2	0	0	0	0	0	0	1	1	0	1	1
hsa-miR-152	MIMAT0000438	NM_014924	KIAA0831	KIAA0831	0	0	1	0	0	0	1	1	0	1	1
hsa-miR-152	MIMAT0000438	NM_014945	ABLIM3	actin binding LIM protein family, member 3	0	0	0	0	0	0	1	1	0	1	1
hsa-miR-152	MIMAT0000438	NM_014962	BTBD3	BTB (POZ) domain containing 3	0	0	1	1	0	0	1	1	0	1	1
hsa-miR-152	MIMAT0000438	NM_014964	EPN2	epsin 2	0	0	1	0	0	0	1	1	0	1	1
hsa-miR-152	MIMAT0000438	NM_014969	WDR47	WD repeat domain 47	0	0	1	1	0	0	0	1	0	1	0
hsa-miR-152	MIMAT0000438	NM_015017	USP33	ubiquitin specific peptidase 33	0	0	0	1	0	0	1	1	0	1	1
hsa-miR-152	MIMAT0000438	NM_015045	WAPAL	wings apart-like homolog (Drosophila)	0	0	0	0	0	0	1	1	0	1	1
hsa-miR-152	MIMAT0000438	NM_015047	KIAA0090	KIAA0090	0	0	1	1	0	0	0	1	0	1	0
hsa-miR-152	MIMAT0000438	NM_015049	TRAK2	trafficking protein, kinesin binding 2	0	0	1	1	0	0	1	1	0	1	1
hsa-miR-152	MIMAT0000438	NM_015050	KIAA0082	KIAA0082	0	0	1	0	0	0	1	1	0	1	1
hsa-miR-152	MIMAT0000438	NM_015070	ZC3H13	zinc finger CCCH-type containing 13	0	0	1	1	0	0	0	1	0	1	0
hsa-miR-152	MIMAT0000438	NM_015076	CDC2L6	cell division cycle 2-like 6 (CDK8-like)	0	0	1	1	0	0	1	1	0	1	1
hsa-miR-152	MIMAT0000438	NM_015088	TNRC6B	trinucleotide repeat containing 6B	0	0	1	1	0	0	0	1	0	1	0
hsa-miR-152	MIMAT0000438	NM_015115	DCUN1D4	DCN1, defective in cullin neddylation 1, domain containing 4 (S. cerevisiae)	0	0	1	1	0	0	0	1	0	1	0
hsa-miR-152	MIMAT0000438	NM_015153	PHF3	PHD finger protein 3	0	0	1	1	0	0	1	1	0	1	1
hsa-miR-152	MIMAT0000438	NM_015161	ARL6IP1	ADP-ribosylation factor-like 6 interacting protein 1	0	0	1	1	0	0	1	1	0	1	1
hsa-miR-152	MIMAT0000438	NM_015170	SULF1	sulfatase 1	0	0	0	0	0	0	1	1	0	1	1
hsa-miR-152	MIMAT0000438	NM_015176	FBXO28	F-box protein 28	0	0	1	1	0	0	0	1	0	1	0
hsa-miR-152	MIMAT0000438	NM_015200	PDS5A	PDS5, regulator of cohesion maintenance, homolog A (S. cerevisiae)	0	0	1	1	0	0	0	1	0	1	0
hsa-miR-152	MIMAT0000438	NM_015205	ATP11A	ATPase, class VI, type 11A	0	0	1	1	0	0	0	1	0	1	0

hsa-miR-152	MIMAT0000438	NM_015234	GPR116	G protein-coupled receptor 116	0	0	0	1	0	0	1	1	0	1	1
hsa-miR-152	MIMAT0000438	NM_015271	TRIM2	tripartite motif-containing 2	0	0	0	1	0	0	1	1	0	1	1
hsa-miR-152	MIMAT0000438	NM_015288	PHF15	PHD finger protein 15	0	0	0	0	0	0	1	1	0	1	1
hsa-miR-152	MIMAT0000438	NM_015296	DOCK9	dedicator of cytokinesis 9	0	0	1	0	0	0	0	1	0	1	1
hsa-miR-152	MIMAT0000438	NM_015313	ARHGEF12	Rho guanine nucleotide exchange factor (GEF) 12	0	0	1	0	0	0	1	1	0	1	1
hsa-miR-152	MIMAT0000438	NM_015336	ZDHHC17	zinc finger, DHHC-type containing 17	0	0	1	1	0	0	1	1	0	1	1
hsa-miR-152	MIMAT0000438	NM_015344	LEPROTL1	leptin receptor overlapping transcript-like 1	0	0	1	1	0	0	0	1	0	1	0
hsa-miR-152	MIMAT0000438	NM_015346	ZFYVE26	zinc finger, FYVE domain containing 26	0	0	0	1	0	0	0	1	0	1	1
hsa-miR-152	MIMAT0000438	NM_015443	KIAA1267	KIAA1267	0	0	1	0	0	0	1	1	0	1	1
hsa-miR-152	MIMAT0000438	NM_015455	CNOT6	CCR4-NOT transcription complex, subunit 6	0	0	1	0	0	0	1	1	0	1	1
hsa-miR-152	MIMAT0000438	NM_015458	MTMR9	myotubularin related protein 9	0	0	1	1	0	0	0	1	0	1	0
hsa-miR-152	MIMAT0000438	NM_015560	OPA1	optic atrophy 1 (autosomal dominant)	0	0	1	1	0	0	1	1	0	1	0
hsa-miR-152	MIMAT0000438	NM_015578	LSM14A	LSM14A, SCD6 homolog A (S. cerevisiae)	0	0	0	0	0	0	1	1	0	1	1
hsa-miR-152	MIMAT0000438	NM_015609	C1orf144	chromosome 1 open reading frame 144	0	0	0	1	0	0	1	1	0	1	1
hsa-miR-152	MIMAT0000438	NM_015635	GAPVD1	GTPase activating protein and VPS9 domains 1	0	0	1	1	0	0	0	1	0	1	0
hsa-miR-152	MIMAT0000438	NM_015640	SERBP1	SERPINE1 mRNA binding protein 1	0	0	1	1	0	0	0	1	0	1	0
hsa-miR-152	MIMAT0000438	NM_015678	NBEA	neurobeachin	0	0	0	0	0	0	1	1	0	1	1
hsa-miR-152	MIMAT0000438	NM_015853	LOC51035	SAPK substrate protein 1	0	0	0	0	0	0	1	1	0	1	1
hsa-miR-152	MIMAT0000438	NM_015879	ST8SIA3	ST8 alpha-N-acetyl-neuraminide alpha-2,8-sialyltransferase 3	0	0	1	1	0	0	1	1	0	1	1
hsa-miR-152	MIMAT0000438	NM_015898	ZBTB7A	zinc finger and BTB domain containing 7A	0	0	1	0	0	0	1	1	0	1	1
hsa-miR-152	MIMAT0000438	NM_015981	CAMK2A	calcium/calmodulin-dependent protein kinase (CaM kinase) II alpha	0	0	1	0	0	0	1	1	0	1	1
hsa-miR-152	MIMAT0000438	NM_015990	KLHL5	kelch-like 5 (Drosophila)	0	0	1	1	0	0	0	1	0	1	0
hsa-miR-152	MIMAT0000438	NM_016072	GOLT1B	golgi transport 1 homolog B (S. cerevisiae)	0	0	1	1	0	0	0	1	0	1	0
hsa-miR-152	MIMAT0000438	NM_016120	RNF12	ring finger protein 12	0	0	0	1	0	0	0	1	0	1	1
hsa-miR-152	MIMAT0000438	NM_016124	RHD	Rh blood group, D antigen	0	0	1	1	0	0	0	1	0	1	0
hsa-miR-152	MIMAT0000438	NM_016203	PRKAG2	protein kinase, AMP-activated, gamma 2 non-catalytic subunit	0	0	1	0	0	0	1	1	0	1	0
hsa-miR-152	MIMAT0000438	NM_016230	CYB5R4	cytochrome b5 reductase 4	0	0	1	0	0	0	0	1	0	1	1

hsa-miR-152	MIMAT0000438	NM_016231	NLK	nemo-like kinase	0	0	1	0	0	0	1	1	0	1	1
hsa-miR-152	MIMAT0000438	NM_016235	GPRC5B	G protein-coupled receptor, family C, group 5, member B	0	0	1	0	0	0	1	1	0	1	1
hsa-miR-152	MIMAT0000438	NM_016324	ZNF274	zinc finger protein 274	0	0	1	1	0	0	0	1	0	1	0
hsa-miR-152	MIMAT0000438	NM_016325	ZNF274	zinc finger protein 274	0	0	1	1	0	0	0	1	0	1	0
hsa-miR-152	MIMAT0000438	NM_016356	DCDC2	doublecortin domain containing 2	0	0	1	1	0	0	0	1	0	1	0
hsa-miR-152	MIMAT0000438	NM_016377	AKAP7	A kinase (PRKA) anchor protein 7	0	0	1	0	0	0	1	1	0	1	0
hsa-miR-152	MIMAT0000438	NM_016417	GLRX5	glutaredoxin 5	0	0	0	1	0	0	1	1	0	1	1
hsa-miR-152	MIMAT0000438	NM_016436	PHF20	PHD finger protein 20	0	0	1	0	0	0	0	1	0	1	1
hsa-miR-152	MIMAT0000438	NM_016467	ORMDL1	ORM1-like 1 (S. cerevisiae)	0	0	1	1	0	0	0	1	0	1	0
hsa-miR-152	MIMAT0000438	NM_016496	MARCHF2	membrane-associated ring finger (C3HC4) 2	0	0	0	1	0	0	1	1	0	1	0
hsa-miR-152	MIMAT0000438	NM_016538	SIRT7	sirtuin (silent mating type information regulation 2 homolog) 7 (S. cerevisiae)	0	0	1	0	0	0	1	0	0	1	1
hsa-miR-152	MIMAT0000438	NM_016603	FAM13B1	family with sequence similarity 13, member B1	0	0	1	0	0	0	1	1	0	1	1
hsa-miR-152	MIMAT0000438	NM_016626	MEX3C	mex-3 homolog C (C. elegans)	0	0	1	0	0	0	1	1	0	1	1
hsa-miR-152	MIMAT0000438	NM_017455	NPTN	neuroplastin	0	0	1	1	0	0	1	1	0	1	0
hsa-miR-152	MIMAT0000438	NM_017553	INOC1	INO80 complex homolog 1 (S. cerevisiae)	0	0	1	1	0	0	1	1	0	1	1
hsa-miR-152	MIMAT0000438	NM_017626	DNAJB12	DnaJ (Hsp40) homolog, subfamily B, member 12	0	0	1	1	0	0	0	1	0	1	0
hsa-miR-152	MIMAT0000438	NM_017629	EIF2C4	eukaryotic translation initiation factor 2C, 4	0	0	1	1	0	0	1	1	0	1	1
hsa-miR-152	MIMAT0000438	NM_017740	ZDHHC7	zinc finger, DHHC-type containing 7	0	0	1	0	0	0	1	1	0	1	1
hsa-miR-152	MIMAT0000438	NM_017742	ZCCHC2	zinc finger, CCHC domain containing 2	0	0	1	0	0	0	1	1	0	1	1
hsa-miR-152	MIMAT0000438	NM_017761	PNRC2	proline-rich nuclear receptor coactivator 2	0	0	1	1	0	0	0	1	0	1	0
hsa-miR-152	MIMAT0000438	NM_017762	MTMR10	myotubularin related protein 10	0	0	1	1	0	0	0	1	0	1	0
hsa-miR-152	MIMAT0000438	NM_017780	CHD7	chromodomain helicase DNA binding protein 7	0	0	1	1	0	0	1	1	0	1	1
hsa-miR-152	MIMAT0000438	NM_017849	TMEM127	transmembrane protein 127	0	0	0	0	0	0	1	1	0	1	1
hsa-miR-152	MIMAT0000438	NM_017925	DENND4C	DENN/MADD domain containing 4C	0	0	1	1	0	0	0	1	0	1	1
hsa-miR-152	MIMAT0000438	NM_017944	USP47	ubiquitin specific peptidase 47	0	0	1	0	0	0	1	1	0	1	1
hsa-miR-152	MIMAT0000438	NM_018032	LUC7L	LUC7-like (S. cerevisiae)	0	0	1	1	0	0	0	1	0	1	0
hsa-miR-152	MIMAT0000438	NM_018121	C10orf6	chromosome 10 open reading frame 6	0	0	1	0	0	0	1	1	0	1	1

hsa-miR-152	MIMAT0000438	NM_018131	CEP55	centrosomal protein 55kDa	0	0	1	1	0	0	0	1	0	1	0
hsa-miR-152	MIMAT0000438	NM_018184	ARL8B	ADP-ribosylation factor-like 8B	0	0	1	1	0	0	1	1	0	1	1
hsa-miR-152	MIMAT0000438	NM_018226	RNPEPL1	arginyl aminopeptidase (aminopeptidase B)-like 1	0	0	1	0	0	0	1	1	0	1	0
hsa-miR-152	MIMAT0000438	NM_018227	UBA6	ubiquitin-like modifier activating enzyme 6	0	0	1	1	0	0	0	1	0	1	0
hsa-miR-152	MIMAT0000438	NM_018284	GBP3	guanylate binding protein 3	0	0	1	1	0	0	0	1	0	1	0
hsa-miR-152	MIMAT0000438	NM_018299	UBE2W	ubiquitin-conjugating enzyme E2W (putative)	0	0	1	0	0	0	1	1	0	1	0
hsa-miR-152	MIMAT0000438	NM_018327	SPTLC3	serine palmitoyltransferase, long chain base subunit 3	0	0	1	1	0	0	0	1	0	1	0
hsa-miR-152	MIMAT0000438	NM_018426	TMEM63B	transmembrane protein 63B	0	0	0	1	0	0	0	1	0	1	1
hsa-miR-152	MIMAT0000438	NM_018448	CAND1	cullin-associated and neddylation-dissociated 1	0	0	0	1	0	0	1	1	0	1	1
hsa-miR-152	MIMAT0000438	NM_018571	ALS2CR2	amyotrophic lateral sclerosis 2 (juvenile) chromosome region, candidate 2	0	0	1	0	0	0	1	1	0	1	1
hsa-miR-152	MIMAT0000438	NM_018948	ERRFI1	ERBB receptor feedback inhibitor 1	0	0	1	1	0	0	1	1	0	1	1
hsa-miR-152	MIMAT0000438	NM_019556	MOSPD1	motile sperm domain containing 1	0	0	1	1	0	0	1	1	0	1	1
hsa-miR-152	MIMAT0000438	NM_019590	KIAA1217	KIAA1217	0	0	0	0	0	0	1	1	0	1	1
hsa-miR-152	MIMAT0000438	NM_019593	RP5-1022P6.2	hypothetical protein KIAA1434	0	0	1	0	0	0	1	1	0	1	1
hsa-miR-152	MIMAT0000438	NM_019595	ITSN2	intersectin 2	0	0	0	1	0	0	1	1	0	1	0
hsa-miR-152	MIMAT0000438	NM_020128	MDM1	Mdm1 nuclear protein homolog (mouse)	0	0	1	1	0	0	0	1	0	1	0
hsa-miR-152	MIMAT0000438	NM_020156	C1GALT1	core 1 synthase, glycoprotein-N-acetylgalactosamine 3-beta-galactosyltransferase, 1	0	0	1	1	0	0	1	1	0	1	1
hsa-miR-152	MIMAT0000438	NM_020211	RGMA	RGM domain family, member A	0	0	1	0	0	0	1	1	0	1	1
hsa-miR-152	MIMAT0000438	NM_020310	MNT	MAX binding protein	0	0	1	1	0	0	1	1	0	1	1
hsa-miR-152	MIMAT0000438	NM_020378	NAT14	N-acetyltransferase 14	0	0	1	0	0	0	0	1	0	1	1
hsa-miR-152	MIMAT0000438	NM_020473	PIGA	phosphatidylinositol glycan anchor biosynthesis, class A (paroxysmal nocturnal hemoglobinuria)	0	0	1	1	0	0	0	1	0	1	0
hsa-miR-152	MIMAT0000438	NM_020524	PBXIP1	pre-B-cell leukemia homeobox interacting protein 1	0	0	1	0	0	0	1	1	0	1	1
hsa-miR-152	MIMAT0000438	NM_020526	EPHA8	EPH receptor A8	0	0	1	0	0	0	1	1	0	1	1
hsa-miR-152	MIMAT0000438	NM_020536	CSRP2BP	CSRP2 binding protein	0	0	0	0	0	0	1	1	0	1	1
hsa-miR-152	MIMAT0000438	NM_020546	ADCY2	adenylate cyclase 2 (brain)	0	0	1	0	0	0	1	1	0	1	1
hsa-miR-152	MIMAT0000438	NM_020644	TMEM9B	TMEM9 domain family, member B	0	0	1	1	0	0	1	1	0	1	1
hsa-miR-152	MIMAT0000438	NM_020655	JPH3	junctophilin 3	0	0	1	1	0	0	1	1	0	1	1

hsa-miR-152	MIMAT0000438	NM_020689	SLC24A3	solute carrier family 24 (sodium/potassium/calcium exchanger), member 3	0	0	1	1	0	0	1	1	0	1	1
hsa-miR-152	MIMAT0000438	NM_020718	USP31	ubiquitin specific peptidase 31	0	0	1	1	0	0	0	1	0	1	0
hsa-miR-152	MIMAT0000438	NM_020760	HECW2	HECT, C2 and WW domain containing E3 ubiquitin protein ligase 2	0	0	1	0	0	0	0	1	0	1	1
hsa-miR-152	MIMAT0000438	NM_020801	ARRDC3	arrestin domain containing 3	0	0	0	1	0	0	1	1	0	1	1
hsa-miR-152	MIMAT0000438	NM_020809	ARHGAP20	Rho GTPase activating protein 20	0	0	0	0	0	0	1	1	0	1	1
hsa-miR-152	MIMAT0000438	NM_020812	DOCK6	dedicator of cytokinesis 6	0	0	1	0	0	0	1	1	0	1	1
hsa-miR-152	MIMAT0000438	NM_020824	ARHGAP21	Rho GTPase activating protein 21	0	0	1	1	0	0	1	1	0	1	1
hsa-miR-152	MIMAT0000438	NM_020826	SYT13	synaptotagmin XIII	0	0	1	1	0	0	0	1	0	1	0
hsa-miR-152	MIMAT0000438	NM_020854	KIAA1468	KIAA1468	0	0	0	1	0	0	1	1	0	1	1
hsa-miR-152	MIMAT0000438	NM_020909	EPB41L5	erythrocyte membrane protein band 4.1 like 5	0	0	1	1	0	0	0	1	0	1	0
hsa-miR-152	MIMAT0000438	NM_020948	MIER1	mesoderm induction early response 1 homolog (Xenopus laevis)	0	0	1	1	0	0	1	1	0	1	0
hsa-miR-152	MIMAT0000438	NM_020962	NOPE	neighbor of Punc E11	0	0	0	0	0	0	1	1	0	1	1
hsa-miR-152	MIMAT0000438	NM_020964	KIAA1632	KIAA1632	0	0	1	1	0	0	0	1	0	1	1
hsa-miR-152	MIMAT0000438	NM_020977	ANK2	ankyrin 2, neuronal	0	0	1	0	0	0	1	1	0	1	0
hsa-miR-152	MIMAT0000438	NM_020993	BCL7A	B-cell CLL/lymphoma 7A	0	0	1	0	0	0	1	1	0	1	0
hsa-miR-152	MIMAT0000438	NM_021109	TMSB4X	thymosin beta 4, X-linked	0	0	1	0	0	0	1	1	0	1	1
hsa-miR-152	MIMAT0000438	NM_021728	OTX2	orthodenticle homeobox 2	0	0	1	1	0	0	1	1	0	1	1
hsa-miR-152	MIMAT0000438	NM_021809	TGIF2	TGFB-induced factor homeobox 2	0	0	1	1	0	0	1	1	0	1	1
hsa-miR-152	MIMAT0000438	NM_021813	BACH2	BTB and CNC homology 1, basic leucine zipper transcription factor 2	0	0	1	0	0	0	1	1	0	1	1
hsa-miR-152	MIMAT0000438	NM_021815	SLC5A7	solute carrier family 5 (choline transporter), member 7	0	0	1	1	0	0	0	1	0	1	0
hsa-miR-152	MIMAT0000438	NM_021914	CFL2	cofilin 2 (muscle)	0	0	1	1	0	0	1	1	0	1	1
hsa-miR-152	MIMAT0000438	NM_021961	TEAD1	TEA domain family member 1 (SV40 transcriptional enhancer factor)	0	0	1	0	0	0	1	1	0	1	1
hsa-miR-152	MIMAT0000438	NM_022459	XPO4	exportin 4	0	0	1	1	0	0	1	1	0	1	1
hsa-miR-152	MIMAT0000438	NM_022485	MTMR14	myotubularin related protein 14	0	0	1	0	0	0	1	1	0	1	0
hsa-miR-152	MIMAT0000438	NM_022658	HOXC8	homeobox C8	0	0	1	1	0	0	1	1	0	1	1
hsa-miR-152	MIMAT0000438	NM_022776	OSBPL11	oxysterol binding protein-like 11	0	0	1	1	0	0	1	1	0	1	1
hsa-miR-152	MIMAT0000438	NM_022780	RMND5A	required for meiotic nuclear division 5 homolog A (S. cerevisiae)	0	0	0	0	0	0	1	1	0	1	1

hsa-miR-152	MIMAT0000438	NM_022781	RNF38	ring finger protein 38	0	0	1	1	0	0	1	1	0	1	1
hsa-miR-152	MIMAT0000438	NM_022822	KLC2	kinesin light chain 2	0	0	1	0	0	0	1	1	0	1	1
hsa-miR-152	MIMAT0000438	NM_022893	BCL11A	B-cell CLL/lymphoma 11A (zinc finger protein)	0	0	1	0	0	0	1	1	0	1	1
hsa-miR-152	MIMAT0000438	NM_022903	CCDC71	coiled-coil domain containing 71	0	0	0	1	0	0	0	1	0	1	1
hsa-miR-152	MIMAT0000438	NM_024119	DHX58	DEXH (Asp-Glu-X-His) box polypeptide 58	0	0	1	1	0	0	0	1	0	1	0
hsa-miR-152	MIMAT0000438	NM_024422	DSC2	desmocollin 2	0	0	1	1	0	0	0	1	0	1	0
hsa-miR-152	MIMAT0000438	NM_024581	C6orf60	chromosome 6 open reading frame 60	0	0	1	1	0	0	0	1	0	1	0
hsa-miR-152	MIMAT0000438	NM_024629	MLF1IP	MLF1 interacting protein	0	0	1	1	0	0	0	1	0	1	0
hsa-miR-152	MIMAT0000438	NM_024663	NPEPL1	aminopeptidase-like 1	0	0	0	1	0	0	1	1	0	1	1
hsa-miR-152	MIMAT0000438	NM_024665	TBL1XR1	transducin (beta)-like 1 X-linked receptor 1	0	0	1	1	0	0	0	1	0	1	1
hsa-miR-152	MIMAT0000438	NM_024674	LIN28	lin-28 homolog (C. elegans)	0	0	1	0	0	0	1	1	0	1	1
hsa-miR-152	MIMAT0000438	NM_024841	FLJ14213	protor-2	0	0	1	0	0	0	1	1	0	1	1
hsa-miR-152	MIMAT0000438	NM_024896	ERMP1	endoplasmic reticulum metallopeptidase 1	0	0	1	1	0	0	0	1	0	1	0
hsa-miR-152	MIMAT0000438	NM_025059	C6orf97	chromosome 6 open reading frame 97	0	0	1	1	0	0	0	1	0	1	0
hsa-miR-152	MIMAT0000438	NM_025133	FBXO11	F-box protein 11	0	0	0	1	0	0	1	1	0	1	1
hsa-miR-152	MIMAT0000438	NM_025134	CHD9	chromodomain helicase DNA binding protein 9	0	0	1	1	0	0	1	1	0	1	1
hsa-miR-152	MIMAT0000438	NM_025180	CEP63	centrosomal protein 63kDa	0	0	1	1	0	0	0	1	0	1	0
hsa-miR-152	MIMAT0000438	NM_025187	C16orf70	chromosome 16 open reading frame 70	0	0	1	0	0	0	1	1	0	1	1
hsa-miR-152	MIMAT0000438	NM_025222	WDR82	WD repeat domain 82	0	0	1	0	0	0	0	1	0	1	1
hsa-miR-152	MIMAT0000438	NM_030576	LIMD2	LIM domain containing 2	0	0	1	0	0	0	1	1	0	1	1
hsa-miR-152	MIMAT0000438	NM_030581	WDR59	WD repeat domain 59	0	0	1	1	0	0	0	1	0	1	0
hsa-miR-152	MIMAT0000438	NM_030621	DICER1	dicer 1, ribonuclease type III	0	0	1	0	0	0	1	1	0	1	1
hsa-miR-152	MIMAT0000438	NM_030627	CPEB4	cytoplasmic polyadenylation element binding protein 4	0	0	1	0	0	0	1	1	0	1	1
hsa-miR-152	MIMAT0000438	NM_030918	SNX27	sorting nexin family member 27	0	0	1	0	0	0	1	1	0	1	1
hsa-miR-152	MIMAT0000438	NM_030938	TMEM49	transmembrane protein 49	0	0	1	1	0	0	0	1	0	1	0
hsa-miR-152	MIMAT0000438	NM_030939	C6orf62	chromosome 6 open reading frame 62	0	0	1	0	0	0	1	1	0	1	1
hsa-miR-152	MIMAT0000438	NM_030962	SBF2	SET binding factor 2	0	0	1	0	0	0	1	1	0	1	1

hsa-miR-152	MIMAT0000438	NM_031477	YPEL3	yippee-like 3 (Drosophila)	0	0	1	0	0	0	1	1	0	1	1
hsa-miR-152	MIMAT0000438	NM_031934	RAB34	RAB34, member RAS oncogene family	0	0	0	1	0	0	1	1	0	1	1
hsa-miR-152	MIMAT0000438	NM_032039	ITFG3	integrin alpha FG-GAP repeat containing 3	0	0	1	1	0	0	1	1	0	1	1
hsa-miR-152	MIMAT0000438	NM_032177	RNUXA	RNA U, small nuclear RNA export adaptor (phosphorylation regulated)	0	0	1	1	0	0	0	1	0	1	0
hsa-miR-152	MIMAT0000438	NM_032189	ATP11A	ATPase, class VI, type 11A	0	0	1	1	0	0	0	1	0	1	0
hsa-miR-152	MIMAT0000438	NM_032236	USP48	ubiquitin specific peptidase 48	0	0	1	1	0	0	1	1	0	1	1
hsa-miR-152	MIMAT0000438	NM_032268	ZNRF1	zinc and ring finger 1	0	0	1	0	0	0	1	1	0	1	1
hsa-miR-152	MIMAT0000438	NM_032272	MAF1	MAF1 homolog (S. cerevisiae)	0	0	0	0	0	0	1	1	0	1	1
hsa-miR-152	MIMAT0000438	NM_032320	BTBD10	BTB (POZ) domain containing 10	0	0	0	0	0	0	1	1	0	1	1
hsa-miR-152	MIMAT0000438	NM_032582	USP32	ubiquitin specific peptidase 32	0	0	0	1	0	0	1	1	0	1	1
hsa-miR-152	MIMAT0000438	NM_032595	PPP1R9B	protein phosphatase 1, regulatory (inhibitor) subunit 9B	0	0	1	0	0	0	0	1	0	1	1
hsa-miR-152	MIMAT0000438	NM_032837	FAM104A	family with sequence similarity 104, member A	0	0	1	1	0	0	1	1	0	1	1
hsa-miR-152	MIMAT0000438	NM_032876	JUB	jub, ajuba homolog (Xenopus laevis)	0	0	1	0	0	0	0	1	0	1	1
hsa-miR-152	MIMAT0000438	NM_033141	MAP3K9	mitogen-activated protein kinase kinase kinase 9	0	0	1	0	0	0	1	1	0	1	1
hsa-miR-152	MIMAT0000438	NM_033150	COL2A1	collagen, type II, alpha 1	0	0	1	1	0	0	1	1	0	1	0
hsa-miR-152	MIMAT0000438	NM_033211	C5orf30	chromosome 5 open reading frame 30	0	0	1	1	0	0	1	1	0	1	1
hsa-miR-152	MIMAT0000438	NM_033224	PURB	purine-rich element binding protein B	0	0	1	0	0	0	1	1	0	1	1
hsa-miR-152	MIMAT0000438	NM_033300	LRP8	low density lipoprotein receptor-related protein 8, apolipoprotein e receptor	0	0	1	0	0	0	1	1	0	1	1
hsa-miR-152	MIMAT0000438	NM_052851	STARD13	StAR-related lipid transfer (START) domain containing 13	0	0	1	0	0	0	1	1	0	1	1
hsa-miR-152	MIMAT0000438	NM_052925	LENG8	leukocyte receptor cluster (LRC) member 8	0	0	0	0	0	0	1	1	0	1	1
hsa-miR-152	MIMAT0000438	NM_053002	MED12L	mediator complex subunit 12-like	0	0	1	1	0	0	1	1	0	1	1
hsa-miR-152	MIMAT0000438	NM_054031	MRGPRX3	MAS-related GPR, member X3	0	0	1	1	0	0	0	1	0	1	0
hsa-miR-152	MIMAT0000438	NM_057175	NARG1	NMDA receptor regulated 1	0	0	1	1	0	0	1	1	0	1	1
hsa-miR-152	MIMAT0000438	NM_080677	DYNLL2	dynein, light chain, LC8-type 2	0	0	0	1	0	0	1	1	0	1	1
hsa-miR-152	MIMAT0000438	NM_101395	DYRK1A	dual-specificity tyrosine-(Y)-phosphorylation regulated kinase 1A	0	0	1	0	0	0	1	1	0	1	1
hsa-miR-152	MIMAT0000438	NM_130436	DYRK1A	dual-specificity tyrosine-(Y)-phosphorylation regulated kinase 1A	0	0	1	0	0	0	1	1	0	1	0
hsa-miR-152	MIMAT0000438	NM_130437	DYRK1A	dual-specificity tyrosine-(Y)-phosphorylation regulated kinase 1A	0	0	1	0	0	0	1	1	0	1	0

hsa-miR-152	MIMAT0000438	NM_130438	DYRK1A	dual-specificity tyrosine-(Y)-phosphorylation regulated kinase 1A	0	0	1	0	0	0	1	1	0	1	0
hsa-miR-152	MIMAT0000438	NM_130442	ELMO1	engulfment and cell motility 1	0	0	1	0	0	0	1	1	0	1	0
hsa-miR-152	MIMAT0000438	NM_130443	DPP3	dipeptidyl-peptidase 3	0	0	1	0	0	0	1	1	0	1	0
hsa-miR-152	MIMAT0000438	NM_130806	RXFP2	relaxin/insulin-like family peptide receptor 2	0	0	0	0	0	0	1	1	0	1	1
hsa-miR-152	MIMAT0000438	NM_130831	OPA1	optic atrophy 1 (autosomal dominant)	0	0	1	1	0	0	1	1	0	1	0
hsa-miR-152	MIMAT0000438	NM_130832	OPA1	optic atrophy 1 (autosomal dominant)	0	0	0	1	0	0	1	1	0	1	0
hsa-miR-152	MIMAT0000438	NM_130833	OPA1	optic atrophy 1 (autosomal dominant)	0	0	1	1	0	0	1	1	0	1	0
hsa-miR-152	MIMAT0000438	NM_130834	OPA1	optic atrophy 1 (autosomal dominant)	0	0	1	1	0	0	1	1	0	1	0
hsa-miR-152	MIMAT0000438	NM_130835	OPA1	optic atrophy 1 (autosomal dominant)	0	0	1	1	0	0	1	1	0	1	0
hsa-miR-152	MIMAT0000438	NM_130836	OPA1	optic atrophy 1 (autosomal dominant)	0	0	0	1	0	0	1	1	0	1	0
hsa-miR-152	MIMAT0000438	NM_130837	OPA1	optic atrophy 1 (autosomal dominant)	0	0	0	1	0	0	1	1	0	1	0
hsa-miR-152	MIMAT0000438	NM_133330	WHSC1	Wolf-Hirschhorn syndrome candidate 1	0	0	1	0	0	0	1	1	0	1	0
hsa-miR-152	MIMAT0000438	NM_133331	WHSC1	Wolf-Hirschhorn syndrome candidate 1	0	0	1	0	0	0	1	1	0	1	0
hsa-miR-152	MIMAT0000438	NM_133335	WHSC1	Wolf-Hirschhorn syndrome candidate 1	0	0	1	0	0	0	1	1	0	1	0
hsa-miR-152	MIMAT0000438	NM_133374	ZNF618	zinc finger protein 618	0	0	1	0	0	0	0	1	0	1	1
hsa-miR-152	MIMAT0000438	NM_133631	ROBO1	roundabout, axon guidance receptor, homolog 1 (Drosophila)	0	0	1	1	0	0	1	1	0	1	0
hsa-miR-152	MIMAT0000438	NM_138346	KIAA2013	KIAA2013	0	0	0	0	0	0	1	1	0	1	1
hsa-miR-152	MIMAT0000438	NM_138621	BCL2L11	BCL2-like 11 (apoptosis facilitator)	0	0	1	1	0	0	0	1	0	1	0
hsa-miR-152	MIMAT0000438	NM_138633	AKAP7	A kinase (PRKA) anchor protein 7	0	0	1	0	0	0	1	1	0	1	0
hsa-miR-152	MIMAT0000438	NM_138638	CFL2	cofilin 2 (muscle)	0	0	1	1	0	0	1	1	0	1	0
hsa-miR-152	MIMAT0000438	NM_138714	NFAT5	nuclear factor of activated T-cells 5, tonicity-responsive	0	0	1	0	0	0	1	1	0	1	0
hsa-miR-152	MIMAT0000438	NM_139071	SMARCD1	SWI/SNF related, matrix associated, actin dependent regulator of chromatin, subfamily d, member 1	0	0	1	0	0	0	1	1	0	1	0
hsa-miR-152	MIMAT0000438	NM_139072	DNER	delta/notch-like EGF repeat containing	0	0	0	0	0	0	1	1	0	1	1
hsa-miR-152	MIMAT0000438	NM_139245	PPM1L	protein phosphatase 1 (formerly 2C)-like	0	0	1	1	0	0	0	1	0	1	0
hsa-miR-152	MIMAT0000438	NM_139323	YWHAB	tyrosine 3-monooxygenase/tryptophan 5-monooxygenase activation protein, beta polypeptide	0	0	1	1	0	0	1	1	0	1	0
hsa-miR-152	MIMAT0000438	NM_144599	NIPA1	non imprinted in Prader-Willi/Angelman syndrome 1	0	0	1	1	0	0	0	1	0	1	0
hsa-miR-152	MIMAT0000438	NM_145055	C18orf25	chromosome 18 open reading frame 25	0	0	1	1	0	0	1	1	0	1	0

hsa-miR-152	MIMAT0000438	NM_145113	MAX	MYC associated factor X	0	0	1	0	0	0	1	1	0	1	0
hsa-miR-152	MIMAT0000438	NM_145176	SLC2A12	solute carrier family 2 (facilitated glucose transporter), member 12	0	0	1	1	0	0	0	1	0	1	0
hsa-miR-152	MIMAT0000438	NM_145316	C6orf128	chromosome 6 open reading frame 128	0	0	1	1	0	0	0	1	0	1	0
hsa-miR-152	MIMAT0000438	NM_147156	SGMS1	sphingomyelin synthase 1	0	0	0	0	0	0	1	1	0	1	1
hsa-miR-152	MIMAT0000438	NM_147190	LASS5	LAG1 homolog, ceramide synthase 5	0	0	0	0	0	0	1	1	0	1	1
hsa-miR-152	MIMAT0000438	NM_147223	NCOA1	nuclear receptor coactivator 1	0	0	0	1	0	0	0	1	0	1	1
hsa-miR-152	MIMAT0000438	NM_148921	EPN2	epsin 2	0	0	1	0	0	0	1	1	0	1	0
hsa-miR-152	MIMAT0000438	NM_152264	SLC39A13	solute carrier family 39 (zinc transporter), member 13	0	0	0	0	0	0	1	1	0	1	1
hsa-miR-152	MIMAT0000438	NM_152335	C15orf27	chromosome 15 open reading frame 27	0	0	0	0	0	0	1	1	0	1	1
hsa-miR-152	MIMAT0000438	NM_152409	C5orf24	chromosome 5 open reading frame 24	0	0	1	1	0	0	0	1	0	1	0
hsa-miR-152	MIMAT0000438	NM_152423	MUM1L1	melanoma associated antigen (mutated) 1-like 1	0	0	0	1	0	0	1	1	0	1	1
hsa-miR-152	MIMAT0000438	NM_152470	RNF165	ring finger protein 165	0	0	1	0	0	0	1	1	0	1	1
hsa-miR-152	MIMAT0000438	NM_152553	RNF217	ring finger protein 217	0	0	0	0	0	0	1	1	0	1	1
hsa-miR-152	MIMAT0000438	NM_152556	FLJ31818	hypothetical protein FLJ31818	0	0	1	0	0	0	1	1	0	1	1
hsa-miR-152	MIMAT0000438	NM_152624	DCP2	DCP2 decapping enzyme homolog (S. cerevisiae)	0	0	1	1	0	0	0	1	0	1	1
hsa-miR-152	MIMAT0000438	NM_152716	PATL1	protein associated with topoisomerase II homolog 1 (yeast)	0	0	1	0	0	0	1	1	0	1	1
hsa-miR-152	MIMAT0000438	NM_152748	KIAA1324L	KIAA1324-like	0	0	1	0	0	0	1	1	0	1	1
hsa-miR-152	MIMAT0000438	NM_152878	MAFF	v-maf musculoaponeurotic fibrosarcoma oncogene homolog F (avian)	0	0	1	1	0	0	0	1	0	1	0
hsa-miR-152	MIMAT0000438	NM_153020	RBM24	RNA binding motif protein 24	0	0	1	1	0	0	1	1	0	1	1
hsa-miR-152	MIMAT0000438	NM_153022	C12orf59	chromosome 12 open reading frame 59	0	0	1	1	0	0	0	1	0	1	0
hsa-miR-152	MIMAT0000438	NM_153828	RTN4	reticulon 4	0	0	1	1	0	0	0	1	0	1	0
hsa-miR-152	MIMAT0000438	NM_170665	ATP2A2	ATPase, Ca++ transporting, cardiac muscle, slow twitch 2	0	0	0	0	0	0	1	1	0	1	1
hsa-miR-152	MIMAT0000438	NM_170679	SKP1	S-phase kinase-associated protein 1	0	0	0	1	0	0	1	1	0	1	1
hsa-miR-152	MIMAT0000438	NM_171825	CAMK2A	calcium/calmodulin-dependent protein kinase (CaM kinase) II alpha	0	0	1	0	0	0	1	1	0	1	0
hsa-miR-152	MIMAT0000438	NM_172174	IL15	interleukin 15	0	0	1	1	0	0	0	1	0	1	0
hsa-miR-152	MIMAT0000438	NM_172337	OTX2	orthodenticle homeobox 2	0	0	1	1	0	0	1	1	0	1	0
hsa-miR-152	MIMAT0000438	NM_173060	CAST	calpastatin	0	0	1	1	0	0	0	1	0	1	0

hsa-miR-152	MIMAT0000438	NM_173061	CAST	calpastatin	0	0	1	1	0	0	0	1	0	1	0
hsa-miR-152	MIMAT0000438	NM_173063	CAST	calpastatin	0	0	1	1	0	0	0	1	0	1	0
hsa-miR-152	MIMAT0000438	NM_173084	TRIM59	tripartite motif-containing 59	0	0	1	1	0	0	0	1	0	1	0
hsa-miR-152	MIMAT0000438	NM_173354	SNF1LK	SNF1-like kinase	0	0	1	1	0	0	1	1	0	1	1
hsa-miR-152	MIMAT0000438	NM_173468	MOBKL1A	MOB1, Mps One Binder kinase activator-like 1A (yeast)	0	0	1	1	0	0	0	1	0	1	1
hsa-miR-152	MIMAT0000438	NM_173518	C8orf45	chromosome 8 open reading frame 45	0	0	1	1	0	0	0	1	0	1	0
hsa-miR-152	MIMAT0000438	NM_173531	ZNF100	zinc finger protein 100	0	0	1	1	0	0	0	1	0	1	0
hsa-miR-152	MIMAT0000438	NM_173654	C3orf64	chromosome 3 open reading frame 64	0	0	1	1	0	0	0	1	0	1	0
hsa-miR-152	MIMAT0000438	NM_173797	PAPD4	PAP associated domain containing 4	0	0	1	0	0	0	1	1	0	1	1
hsa-miR-152	MIMAT0000438	NM_174916	UBR1	ubiquitin protein ligase E3 component n-recognin 1	0	0	1	1	0	0	0	1	0	1	1
hsa-miR-152	MIMAT0000438	NM_175607	CNTN4	contactin 4	0	0	1	1	0	0	1	1	0	1	1
hsa-miR-152	MIMAT0000438	NM_175612	CNTN4	contactin 4	0	0	1	1	0	0	1	1	0	1	0
hsa-miR-152	MIMAT0000438	NM_175736	FMNL3	formin-like 3	0	0	0	0	0	0	1	1	0	1	1
hsa-miR-152	MIMAT0000438	NM_175866	UHMK1	U2AF homology motif (UHM) kinase 1	0	0	0	0	0	0	1	1	0	1	1
hsa-miR-152	MIMAT0000438	NM_175907	ZADH2	zinc binding alcohol dehydrogenase domain containing 2	0	0	1	0	0	0	1	1	0	1	1
hsa-miR-152	MIMAT0000438	NM_176824	BBS7	Bardet-Biedl syndrome 7	0	0	1	1	0	0	0	1	0	1	0
hsa-miR-152	MIMAT0000438	NM_176875	CCKBR	cholecystokinin B receptor	0	0	0	1	0	0	1	1	0	1	1
hsa-miR-152	MIMAT0000438	NM_176880	NR2C2AP	nuclear receptor 2C2-associated protein	0	0	1	1	0	0	1	1	0	1	1
hsa-miR-152	MIMAT0000438	NM_177438	DICER1	dicer 1, ribonuclease type III	0	0	1	0	0	0	1	1	0	1	0
hsa-miR-152	MIMAT0000438	NM_178006	STARD13	StAR-related lipid transfer (START) domain containing 13	0	0	1	0	0	0	1	1	0	1	0
hsa-miR-152	MIMAT0000438	NM_178123	SESTD1	SEC14 and spectrin domains 1	0	0	1	1	0	0	1	1	0	1	1
hsa-miR-152	MIMAT0000438	NM_178450	MARCHF3	membrane-associated ring finger (C3HC4) 3	0	0	1	0	0	0	1	1	0	1	1
hsa-miR-152	MIMAT0000438	NM_181291	WDR20	WD repeat domain 20	0	0	0	0	0	0	1	1	0	1	1
hsa-miR-152	MIMAT0000438	NM_181443	BTBD3	BTB (POZ) domain containing 3	0	0	1	1	0	0	1	1	0	1	0
hsa-miR-152	MIMAT0000438	NM_181809	BMP8A	bone morphogenetic protein 8a	0	0	1	1	0	0	0	1	0	1	0
hsa-miR-152	MIMAT0000438	NM_181836	TMED7	transmembrane emp24 protein transport domain containing 7	0	0	0	1	0	0	1	1	0	1	1
hsa-miR-152	MIMAT0000438	NM_182527	CABP7	calcium binding protein 7	0	0	0	1	0	0	1	1	0	1	1

hsa-miR-152	MIMAT0000438	NM_182646	CPEB2	cytoplasmic polyadenylation element binding protein 2	0	0	1	0	0	0	1	1	0	1	0
hsa-miR-152	MIMAT0000438	NM_182760	SUMF1	sulfatase modifying factor 1	0	0	1	1	0	0	0	1	0	1	0
hsa-miR-152	MIMAT0000438	NM_182830	MDGA2	MAM domain containing glycosylphosphatidylinositol anchor 2	0	0	0	0	0	0	1	1	0	1	1
hsa-miR-152	MIMAT0000438	NM_183353	RNF12	ring finger protein 12	0	0	1	1	0	0	0	1	0	1	0
hsa-miR-152	MIMAT0000438	NM_183387	EML5	echinoderm microtubule associated protein like 5	0	0	1	1	0	0	0	1	0	1	0
hsa-miR-152	MIMAT0000438	NM_194071	CREB3L2	cAMP responsive element binding protein 3-like 2	0	0	0	0	0	0	1	1	0	1	1
hsa-miR-152	MIMAT0000438	NM_194328	RNF38	ring finger protein 38	0	0	0	1	0	0	1	1	0	1	0
hsa-miR-152	MIMAT0000438	NM_194329	RNF38	ring finger protein 38	0	0	1	1	0	0	1	1	0	1	0
hsa-miR-152	MIMAT0000438	NM_194330	RNF38	ring finger protein 38	0	0	1	1	0	0	1	1	0	1	0
hsa-miR-152	MIMAT0000438	NM_194332	RNF38	ring finger protein 38	0	0	1	1	0	0	1	1	0	1	0
hsa-miR-152	MIMAT0000438	NM_194442	LBR	lamin B receptor	0	0	1	1	0	0	1	1	0	1	0
hsa-miR-152	MIMAT0000438	NM_197972	NME7	non-metastatic cells 7, protein expressed in (nucleoside-diphosphate kinase)	0	0	1	0	0	0	1	1	0	1	0
hsa-miR-152	MIMAT0000438	NM_198158	MITF	microphthalmia-associated transcription factor	0	0	0	1	0	0	1	1	0	1	0
hsa-miR-152	MIMAT0000438	NM_198159	MITF	microphthalmia-associated transcription factor	0	0	1	1	0	0	1	1	0	1	0
hsa-miR-152	MIMAT0000438	NM_198177	MITF	microphthalmia-associated transcription factor	0	0	1	1	0	0	1	1	0	1	0
hsa-miR-152	MIMAT0000438	NM_198178	MITF	microphthalmia-associated transcription factor	0	0	1	1	0	0	1	1	0	1	0
hsa-miR-152	MIMAT0000438	NM_198270	NHS	Nance-Horan syndrome (congenital cataracts and dental anomalies)	0	0	1	0	0	0	1	1	0	1	1
hsa-miR-152	MIMAT0000438	NM_198273	LYSMD3	LysM, putative peptidoglycan-binding, domain containing 3	0	0	1	1	0	0	0	1	0	1	0
hsa-miR-152	MIMAT0000438	NM_198381	ELF5	E74-like factor 5 (ets domain transcription factor)	0	0	1	0	0	0	1	1	0	1	0
hsa-miR-152	MIMAT0000438	NM_198467	RSBN1L	round spermatid basic protein 1-like	0	0	1	0	0	0	1	1	0	1	1
hsa-miR-152	MIMAT0000438	NM_198715	PTGER3	prostaglandin E receptor 3 (subtype EP3)	0	0	1	1	0	0	0	1	0	1	0
hsa-miR-152	MIMAT0000438	NM_198859	PRICKLE2	prickle homolog 2 (Drosophila)	0	0	1	1	0	0	1	1	0	1	1
hsa-miR-152	MIMAT0000438	NM_199039	KLHL5	kelch-like 5 (Drosophila)	0	0	1	1	0	0	0	1	0	1	0
hsa-miR-152	MIMAT0000438	NM_199072	MDFIC	MyoD family inhibitor domain containing	0	0	0	0	0	0	1	1	0	1	1
hsa-miR-152	MIMAT0000438	NM_199324	OTUD4	OTU domain containing 4	0	0	0	1	0	0	1	1	0	1	1
hsa-miR-152	MIMAT0000438	NM_199355	ADAMTS18	ADAM metallopeptidase with thrombospondin type 1 motif, 18	0	0	0	1	0	0	1	1	0	1	1
hsa-miR-152	MIMAT0000438	NM_201430	RTN3	reticulon 3	0	0	1	1	0	0	0	1	0	1	0

hsa-miR-152	MIMAT0000438	NM_201591	GPM6A	glycoprotein M6A	0	0	0	1	0	0	1	1	0	1	0
hsa-miR-152	MIMAT0000438	NM_201592	GPM6A	glycoprotein M6A	0	0	1	1	0	0	1	1	0	1	0
hsa-miR-152	MIMAT0000438	NM_201624	USP33	ubiquitin specific peptidase 33	0	0	0	1	0	0	1	1	0	1	0
hsa-miR-152	MIMAT0000438	NM_203301	FBXO33	F-box protein 33	0	0	1	1	0	0	1	1	0	1	1
hsa-miR-152	MIMAT0000438	NM_203341	SELENOF	15 kDa selenoprotein	0	0	1	1	0	0	0	1	0	1	0
hsa-miR-152	MIMAT0000438	NM_203374	ZNF784	zinc finger protein 784	0	0	1	1	0	0	0	1	0	1	1
hsa-miR-152	MIMAT0000438	NM_203394	E2F7	E2F transcription factor 7	0	0	1	1	0	0	1	1	0	1	1
hsa-miR-152	MIMAT0000438	NM_205768	ZNF238	zinc finger protein 238	0	0	0	1	0	0	1	1	0	1	0
hsa-miR-152	MIMAT0000438	NM_205848	SYT6	synaptotagmin VI	0	0	1	1	0	0	0	1	0	1	0
hsa-miR-152	MIMAT0000438	NM_206594	ESRRG	estrogen-related receptor gamma	0	0	1	1	0	0	1	1	0	1	0
hsa-miR-152	MIMAT0000438	NM_206595	ESRRG	estrogen-related receptor gamma	0	0	1	1	0	0	1	1	0	1	0
hsa-miR-152	MIMAT0000438	NM_206853	QKI	quaking homolog, KH domain RNA binding (mouse)	0	0	1	1	0	0	1	1	0	1	0
hsa-miR-152	MIMAT0000438	NM_206854	QKI	quaking homolog, KH domain RNA binding (mouse)	0	0	0	1	0	0	1	1	0	1	1
hsa-miR-152	MIMAT0000438	NM_206855	QKI	quaking homolog, KH domain RNA binding (mouse)	0	0	1	1	0	0	1	1	0	1	0
hsa-miR-152	MIMAT0000438	NM_206876	PPP1CB	protein phosphatase 1, catalytic subunit, beta isoform	0	0	1	0	0	0	1	1	0	1	0
hsa-miR-152	MIMAT0000438	NM_207371	C10orf140	chromosome 10 open reading frame 140	0	0	1	1	0	0	1	1	0	1	1
hsa-miR-152	MIMAT0000438	NM_207381	TNFAIP8L3	tumor necrosis factor, alpha-induced protein 8-like 3	0	0	1	1	0	0	0	1	0	1	0
hsa-miR-152	MIMAT0000438	NM_207521	RTN4	reticulon 4	0	0	1	1	0	0	0	1	0	1	0

Table S3. Top significant pathways enriched in predicted miR-152 target genes.

Category	Term	Count	%	*p* Value	Genes	List Total	Pop Hits	Pop Total	Fold Enrichment	Bonferroni	Benjamini	FDR
KEGG_PATHWAY	hsa04350:TGF-beta signaling pathway	12	2.264151	3.03E-05	NM_001429, NM_006930, NM_000627, NM_001105, NM_001106, NM_005406, NM_005450, NM_170679, NM_002193, NM_021809, NM_181809, NM_001001557, NM_001003652	203	84	6879	4.840957	0.006192	0.003549	0.003289
KEGG_PATHWAY	hsa04068:FoxO signaling pathway	15	2.830189	3.46E-05	NM_000314, NM_001924, NM_001429, NM_016231, NM_003629, NM_005544, NM_000618, NM_001400, NM_001556, NM_006538, NM_004272, NM_006251, NM_004064, NM_138621, NM_016203, NM_001040633, NM_001003652	203	134	6879	3.793287	0.007072	0.003549	0.003289
KEGG_PATHWAY	hsa05200:Pathways in cancer	27	5.09434	7.46E-05	NM_001845, NM_003506, NM_000314, NM_002957, NM_001429, NM_003629, NM_001949, NM_000618, NM_020546, NM_006516, NM_005406, NM_000899, NM_003394, NM_004064, NM_198715, NM_003299, NM_005430, NM_198159, NM_198158, NM_198178, NM_198177, NM_001003652, NM_000248, NM_005238, NM_005436, NM_001430, NM_006788, NM_006722, NM_003236, NM_001556, NM_145113, NM_015313	203	393	6879	2.328094	0.015173	0.005096	0.004723
KEGG_PATHWAY	hsa04550:Signaling pathways regulating pluripotency of stem cells	13	2.45283	7.99E-04	NM_003506, NM_003629, NM_004973, NM_004235, NM_014562, NM_000618, NM_001105, NM_001106, NM_002193, NM_003394, NM_006885, NM_005430, NM_001003652	203	140	6879	3.146622	0.151092	0.035624	0.033017
KEGG_PATHWAY	hsa04151:PI3K-Akt signaling pathway	21	3.962264	0.002661	NM_001845, NM_000314, NM_002957, NM_003629, NM_194071, NM_005544, NM_000618, NM_002214, NM_003404, NM_002205, NM_001556, NM_006538, NM_002019, NM_000757, NM_033150, NM_001844, NM_002207, NM_000899, NM_006251, NM_004064,	203	345	6879	2.062669	0.42092	0.068198	0.063208

					NM_138621, NM_139323, NM_003299, NM_001004439							
KEGG_PATHWAY	hsa04390:Hippo signaling pathway	12	2.264151	0.004726	NM_003506, NM_021961, NM_002709, NM_173468, NM_206876, NM_003404, NM_014417, NM_003394, NM_139323, NM_005430, NM_181809, NM_001001557, NM_032876, NM_001003652	203	151	6879	2.692983	0.621357	0.096887	0.089797
KEGG_PATHWAY	hsa04710:Circadian rhythm	5	0.943396	0.012203	NM_170679, NM_006251, NM_016203, NM_006930, NM_004898, NM_006914, NM_001040633	203	31	6879	5.465597	0.919304	0.178691	0.165616
KEGG_PATHWAY	hsa04510:Focal adhesion	13	2.45283	0.017888	NM_001845, NM_000314, NM_003629, NM_002709, NM_000618, NM_002214, NM_206876, NM_002205, NM_002019, NM_033150, NM_001844, NM_002207, NM_005406, NM_002480, NM_001004439	203	206	6879	2.138481	0.975282	0.229189	0.212419
KEGG_PATHWAY	hsa05206:MicroRNAs in cancer	16	3.018868	0.020751	NM_000314, NM_001429, NM_001949, NM_004755, NM_177438, NM_001982, NM_005544, NM_004358, NM_001379, NM_002205, NM_001556, NM_006538, NM_005406, NM_012082, NM_004064, NM_138621, NM_030621, NM_004050	203	286	6879	1.895759	0.986414	0.237857	0.220453
KEGG_PATHWAY	hsa04810:Regulation of actin cytoskeleton	13	2.45283	0.020885	NM_003629, NM_002709, NM_021914, NM_003941, NM_002214, NM_206876, NM_002205, NM_002207, NM_021109, NM_005406, NM_138638, NM_002480, NM_001004439, NM_015313, NM_012219	203	210	6879	2.097748	0.98679	0.237857	0.220453
KEGG_PATHWAY	hsa04066:HIF-1 signaling pathway	8	1.509434	0.022369	NM_001429, NM_004064, NM_003629, NM_015981, NM_003234, NM_000618, NM_002019, NM_006516, NM_171825	203	96	6879	2.823892	0.990319	0.241345	0.223685
KEGG_PATHWAY	hsa04150:mTOR signaling pathway	6	1.132075	0.027052	NM_000314, NM_006251, NM_003629, NM_005544, NM_000618, NM_001556	203	58	6879	3.505521	0.996383	0.264084	0.244761
KEGG_PATHWAY	hsa04110:Cell cycle	9	1.698113	0.0291	NM_170679, NM_001429, NM_001924, NM_004064, NM_001949, NM_139323, NM_003672, NM_006930, NM_004358, NM_003404, NM_001003652	203	124	6879	2.459519	0.997652	0.271159	0.251318
KEGG_PATHWAY	hsa04360:Axon guidance	9	1.698113	0.032917	NM_014945, NM_003873, NM_021914, NM_015313,	203	127	6879	2.40142	0.998953	0.293388	0.27192

					NM_133631, NM_002941, NM_002942, NM_138638, NM_004093, NM_005406, NM_020526							
KEGG_PATHWAY	hsa04114:Oocyte meiosis	8	1.509434	0.044339	NM_170679, NM_182646, NM_139323, NM_002709, NM_015981, NM_006930, NM_000618, NM_206876, NM_003404, NM_030627, NM_020546, NM_171825	203	111	6879	2.442285	0.999908	0.336648	0.312015
KEGG_PATHWAY	hsa04310:Wnt signaling pathway	9	1.698113	0.049796	NM_003506, NM_170679, NM_001429, NM_003394, NM_198859, NM_016231, NM_024665, NM_005430, NM_015981, NM_006930, NM_171825	203	138	6879	2.210002	0.999972	0.364578	0.337901

Publisher's Note: MDPI stays neutral with regard to jurisdictional claims in published maps and institutional affiliations.

© 2020 by the authors. Licensee MDPI, Basel, Switzerland. This article is an open access article distributed under the terms and conditions of the Creative Commons Attribution (CC BY) license (http://creativecommons.org/licenses/by/4.0/).
